# 1,2,3‐Triazole‐Linked Chalcones as Privileged Scaffolds in Anticancer Agents

**DOI:** 10.1002/ardp.70291

**Published:** 2026-07-07

**Authors:** Sumeyya Koldas, Guler Yagiz Erdemir, Ahsen Kilic, Neslisah Barlak, Omer Faruk Karatas, Aliye Altundas

**Affiliations:** ^1^ Department of Chemistry, Faculty of Science Gazi University Ankara Türkiye; ^2^ Department of Chemistry, Graduate School of Natural and Applied Sciences Gazi University Ankara Türkiye; ^3^ Department of Molecular Biology and Genetics Erzurum Technical University Erzurum Türkiye; ^4^ Department of Molecular Cancer Biology Laboratory, High Technology Application and Research Center Erzurum Technical University Erzurum Türkiye

**Keywords:** 1,2,3‐triazolediester, A549, CaCo‐2, cancer, chalcones, cytotoxicity

## Abstract

A new series of extended‐functionality triazole‐substituted chalcone derivatives was synthesized, and their *in vitro* anti‐proliferative and cytotoxic profiles were evaluated using cancer and non‐cancer cell models. First, all compounds were screened in the FaDu head and neck squamous cell carcinoma cell line to identify the most active candidates with comparatively lower IC_50_ values. Compounds **6c**, **6e**, and **6f** reduced FaDu cell viability to 42.62 ± 12.21%, 34.49 ± 4.99%, and 31.13 ± 4.76% of control, respectively. These three compounds also decreased viability of CaCo‐2 human colon cancer cells and A549 human lung adenocarcinoma cells, showing micromolar‐range activity (IC_50_ < 50 µM) across cancer cell lines of different origins. To obtain a preliminary indication of selectivity, we additionally tested a non‐cancer, immortalized prostate epithelial cell line (PNT1a). In this model, **6c**/**6e**/**6f** displayed relatively lower cytotoxicity than in cancer cells, suggesting a preliminary selectivity window within the tested assay framework. Among them, **6f** the most active compound in our series—significantly reduced colony formation, comparable to paclitaxel. In apoptosis‐related assessments, **6f** increased apoptosis‐associated readouts in cancer cells relative to control, although further confirmation is needed with additional orthogonal assays. Overall, our results identify **6c**, **6e**, and especially **6f** as moderately active lead compounds for further optimization of triazole‐chalcone hybrids and support continued structure–activity‐guided development to improve potency and selectivity.

## Graphical abstract

Concise synthesis delivered E‐configured fluoro/methoxy triazole–chalcone hybrids (**6a–i**); structures verified by FT‐IR, 1H/13C NMR, and HRMS. In 24‐h CCK‐8 assays, selected monosubstituted analogues showed low‐micromolar potency against a human lung adenocarcinoma line while sparing non‐malignant PNT1a cells. Structure–activity trends indicate that 3‐position (meta) monosubstitution is beneficial; meta‐OMe and meta‐F motifs consistently enhance activity, nominating **6f** as a tractable lead. Selectivity was consistent across the panel (lung > colorectal > head‐and‐neck) with limited effects on normal cells. The combination of scalable sustainable synthesis, quantitative selectivity, and actionable SAR establishes a clear basis for mechanism‐of‐action and subsequent in vivo studies.

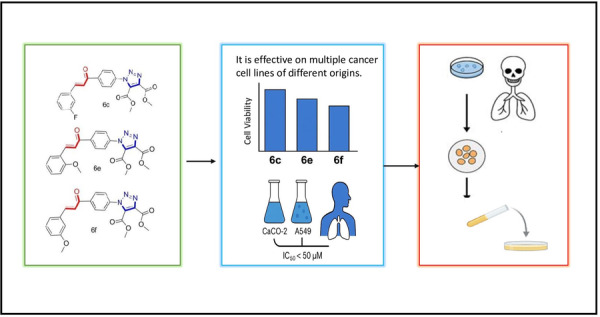

## 1 Introduction

Cancer, one of the most dangerous diseases of the 21st century, threatens the lives of millions of people [1, 2, 3, 4]. Due to increasing environmental risk factors and the aging of societies, the incidence and mortality of cancer continue to increase [5]. Although chemotherapy, radiotherapy, and surgical cancer treatment are the most commonly used methods, cytotoxicity and drug resistance in the treatment process reduce the survival rate of patients [6]. Therefore, there is still a significant need to develop more effective, safe, and affordable therapeutic strategies and tools for the successful treatment of cancer [7, 8].

**Figure 1:**
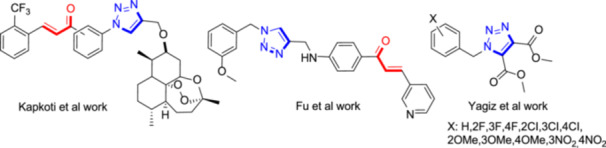
Triazole‐chalcone containing structures.

The biological activities of chalcone compounds can be adjusted by adding various functional groups, such as aryls, halogens, hydroxyls, and carboxyls, which enable chalcones to bind different molecular targets and interact with other molecules [9]. Besides, the hybridization of the chalcone structure with pharmacologically interesting scaffolds to improve therapeutic specificity makes it a promising strategy for developing novel anticancer agents [10]. Triazoles remain a frequently cited scaffold in the literature, especially with the development of click chemistry [11]. These structures are known to be effective motifs in many biological applications and are also found in the structures of many commercial drugs. The triazole ring shares both regularity and angular features with peptide bonds, making these molecules attractive targets in cellular studies [12]. The study of these molecules as effective cores, especially in anticancer screening, has accelerated their modification in this field. It is possible to encounter studies containing many chalcone structures containing triazole rings [13]. Recently, chalcone structures hybridized with triazole skeletons attracted the attention of many scientists as significant anti‐cancer agents in the literature (Figure 1). Kapkoti et al. reported the design of new 1,2,3‐triazole‐chalcone hybrid structures that showed anti‐cancer activity against K562, PC‐3, A431, MDA‐MB‐231, COLO‐205, A549, and HEK‐293 cancer cell lines. Also, these compounds showed no toxicity in regular healthy human cell lines [14]. In another study, Fu et al. investigated the antiproliferative activity of triazole‐containing chalcone derivatives against three cancer cell lines: SK‐N‐SH, HepG‐2, and MGC‐803. They reported that of all hybrids, the molecule as shown in Figure 1 was the best agent with IC_50_ of 1.53 μM against SK‐N‐SH cells [15].

Our group has been working on synthesizing novel triazole derivatives with diverse potential biological activities since 2020 [16], and we have synthesized several lead compounds with distinct biological activities. In a study reported by our group, we showed that triazole molecules containing diester groups exhibit enzyme‐inhibitory potential and effectively inhibit xanthine oxidase. Inspired by the above‐mentioned aspects [15], this study aims to synthesize a new series of triazole‐substituted chalcone derivatives with extended functionality and potential anticancer activity. We evaluated the *in vitro* anti‐cancer and cytotoxic activities of the new chalcone derivatives using different healthy and cancer cell lines, which identified **6c**, **6e**, and especially **6f** as moderately active lead compounds with a preliminary selectivity window within the tested assay framework.

## 2 Results and Discussion

**Scheme 1:**
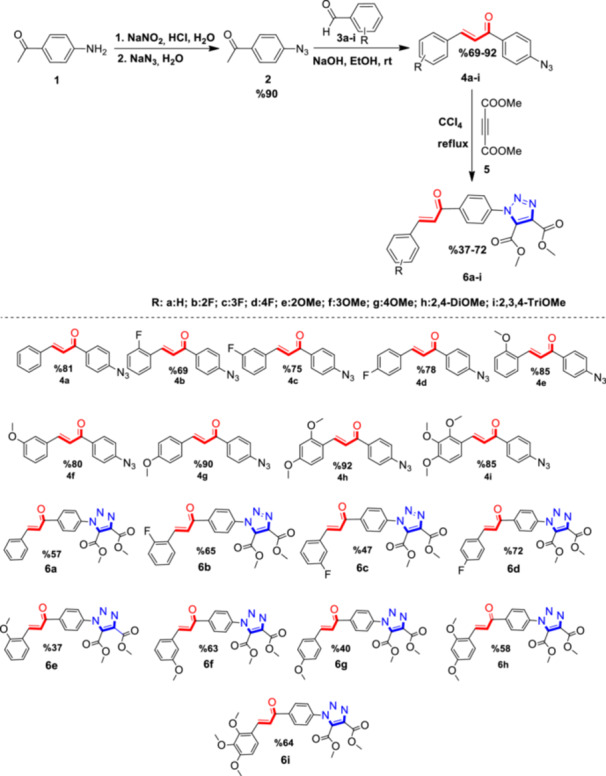
General synthetic pathway of **6a**–**i**.

The synthesis pathway for **6a–i** is illustrated in Scheme 1. The target triazole ring was constructed via a metal‐free [3 + 2] cycloaddition reaction, while the chalcone moiety of the hybrid structures was obtained through a condensation reaction. Initially, the amino group of the 1‐(4‐aminophenyl)ethan‐1‐one was converted into a diazonium salt, an intermediate that enables nucleophilic substitution on the aromatic ring. Then, this diazonium group was subsequently reacted with NaN_3_ to afford 1‐(4‐azidophenyl)ethan‐1‐one (**2**). The obtained azide intermediate was then condensed with various substituted benzaldehydes under basic conditions to yield azide‐containing chalcone derivatives (**4a**–**i**). Finally, the chalcone‐substituted azide was refluxed with DMAD in CCl_4_ to finish the desired 1,2,3‐triazole‐chalcone hybrid structures. The structures of the synthesized hybrids were confirmed by FT‐IR‐ATR, ^1^H and 13 C NMR, and mass spectrometry.

The structure characterization of the synthesized molecules was initially performed by FT‐IR. Compound **2** was confirmed by the appearance of the azide absorption band at approximately 2100 cm^−1^ in the FT‐IR spectrum and the disappearance of the NH_2_ stretching bands around 3400–3300 cm^−1^. Chalcone‐substituted azides were obtained by condensation of 1‐(4‐aminophenyl)ethan‐1‐one with different substituted benzaldehydes (**3a**–**i**) under basic conditions. The structures of the precursor compounds were supported by FT‐IR data, in which the carbonyl stretching band of the conjugated double bond shifted to a lower wavenumber.

In addition, the synthesized molecules were also characterized by ^1^H‐NMR and ^13^C‐NMR. The successful formation of the triazole‐linked chalcone structures was confirmed by the characteristic resonances of the dimethoxy groups attached to the triazole ring at 4.01 and 3.95 ppm in the ^1^H‐NMR spectra, as well as by the C4 and C5 carbon atoms of the triazole ring observed at 132 and 139 ppm, respectively, in the ^13^C‐NMR spectra. Furthermore, HRMS analyses showed that the deviation between the calculated and observed *m*/*z* values of the products was within 5 ppm. Finally, ^1^H‐NMR spectroscopy was used to determine the configuration of the α,β‐unsaturated chalcone moiety in the synthesized derivatives **6a**–**i**. The coupling constants of the olefinic protons, observed in the range of 15–16 Hz, confirmed that all chalcone derivatives adopted the *E* configuration.

In the literature, it is noticeable that fluorine, which is a small atom, and methoxy groups are included in natural product isolations and bioactive derivatives in chalcone chemistry. The fluorine atom is a substituent that draws attention to its therapeutic properties in living tissues. The biological behavior of a molecule can be significantly altered by substituting fluorine for hydrogen. This is frequently caused by fluorine's potent interactions with proteins, which change the selectivity and affinity of binding. The methoxy group can also alter a compound's metabolic stability, making it more resistant to enzyme breakdown and extending its effect in the body, and it is commonly employed to alter the solubility, lipophilicity, and electronic characteristics of molecules. Methoxy groups are essential for fine‐tuning medication potency and selectivity since they can alter receptor binding affinity, improving or decreasing the interaction with target proteins. In medicinal chemistry, methoxy and fluorine atoms are both essential instruments for improving drug candidates' pharmacokinetics, metabolic stability, and receptor interactions, which eventually result in safer and more effective treatments.

In a study by Othman et al., which included the synthesis of 1,2,3‐triazole‐chalcone hybrid molecules to develop new candidates for the treatment of leukemia cancer and the investigation of their anti‐cancer effects, the SAR results reported that especially the electron‐donating groups at C2‐OMe and/or C3‐OMe groups on the benzene ring attached to the enone part of the hybrid structure have an activity‐enhancing effect for all cancer types [16, 17].

**Figure 2:**
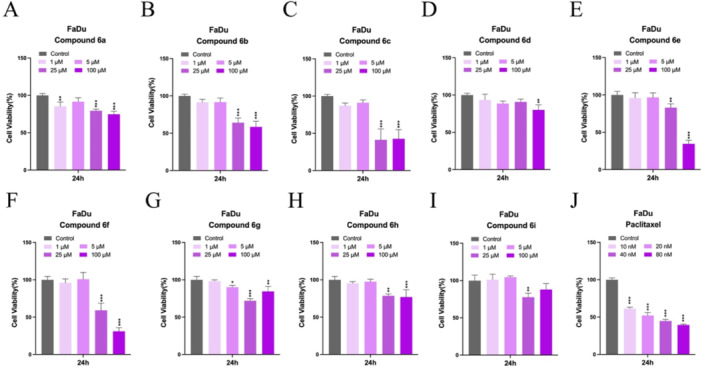
Viability of FaDu cells treated with **6a**–**i** (A–I) and paclitaxel (J) as positive control. *p < 0.05, **p < 0.01, ***p < 0.001; one‐way ANOVA with Dunnett's multiple comparisons test versus control.

Based on this literature, fluorine, and methoxy groups were chosen in the aromatic ring part of the selected hybrid molecules. The synthesized hybrids were screened for effects on cancer cell viability using FaDu cells as an initial model. Paclitaxel served as a positive control. Compounds **6c**, **6e**, and **6f** showed the most pronounced reduction in FaDu viability among the tested set, whereas several compounds showed limited activity at the tested concentrations (Figure 2, Table 1). Compounds **6c**, **6e**, and **6f** reduced FaDu cell viability to 42.62 ± 12.21%, 34.49 ± 4.99%, and 31.13 ± 4.76%, respectively, compared to controls at the highest concentrations.

**Table 1:** IC_50_ values of test compounds on FaDu cells.

	IC_50_ (*µ*M)
Compounds	FaDu cells
**6a**	> 100
**6b**	> 100
**6c**	42.05 ± 6.23
**6d**	> 100
**6e**	79.88 ± 3.99
**6f**	41.12 ± 9.14
**6g**	> 100
**6h**	> 100
**6i**	> 100

**Figure 3:**
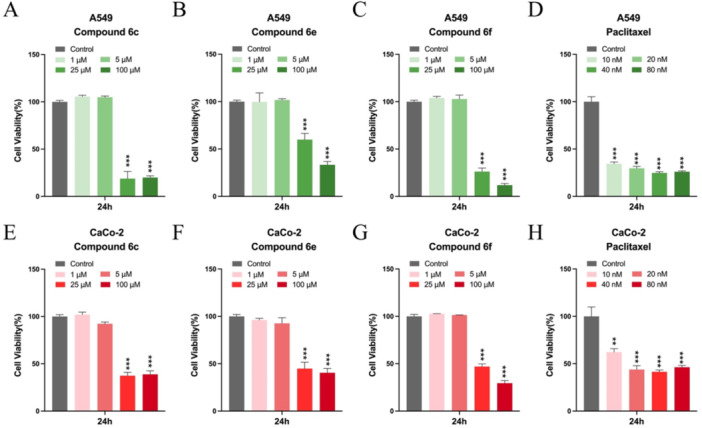
Viability of A549 cell treated with **6c, 6e, 6f** (A–C) and paclitaxel (D) as positive control. Viability of CaCo‐2 cell treated with **6c, 6e, 6f** (E–G), and paclitaxel (H) as a positive control. *p < 0.05, **p < 0.01, ***p < 0.001; one‐way ANOVA with Dunnett's multiple comparisons test versus control.

Then, we evaluated A549 lung adenocarcinoma and CaCo‐2 colon cancer cell lines to assess whether the prioritized compounds (**6c**, **6e**, **6f**) show activity across distinct cancer cell origins. All three compounds reduced viability at higher doses with IC_50_ values in the micromolar range (Figure 3, Table 2).

**Table 2:** IC_50_ values of test compounds on A549, CaCo‐2, and PNT1a cells.

	IC_50_ (*µ*M)		
Compounds	A549	CaCo‐2	PNT1a
**6c**	30.382 ± 0.26	39.86 ± 3.9	55.47 ± 3.60
**6e**	41.049 ± 8.18	47.095 ± 14.69	88.10 ± 1.44
**6f**	11.46 ± 9.87	19.88 ± 4.2	88.66 ± 7.63

**Figure 4:**
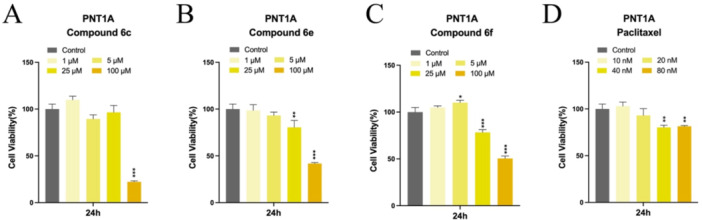
Viability of PNT1a cell treated with **6c, 6e, 6f** (A–C) and Paclitaxel (D) as positive control. *p < 0.05, **p < 0.01, ***p < 0.001; one‐way ANOVA with Dunnett's multiple comparisons test versus control.

A healthy immortalized prostate epithelial cell line (PNT1a) was used as a preliminary model for analysis of cytotoxicity potential of lead compounds. Under the tested conditions, **6c**, **6e**, and **6f** showed higher potency in cancer cell lines than in PNT1a cells with moderate selectivity indices (Figure 4, Table 3) based on the existing IC_50_ estimates similar to the findings of positive control (Table 4), although the selectivity conclusions are preliminary since only one immortalized normal cell model was used.

**Table 3:** Selectivity indices of the prioritized compounds and paclitaxel on FaDu, A549, and CaCo‐2 cells over PNT1a cells.

Cell Line	6c	6e	6f	Paclitaxel
FaDu	1.32	1.1	2.16	5.44
CaCo‐2	1.39	1.87	4.46	6.36
A549	1.83	2.15	7.74	141.5

**Table 4:** IC_50_ values of paclitaxel on FaDu, A549, CaCo‐2, and PNT1a cells.

Cell Line	IC_50_ (nM)
FaDu	26.00 ± 0.01
CaCo‐2	22.25 ± 0.01
A549	1 ± 0.01
PNT1a	141.5 ± 0.03

It is noteworthy that mono‐substituted chalcones tended to be more active than the unsubstituted (**6a**) and the di‐/tri‐methoxy substituted analogs (**6h, 6i**) in the initial FaDu screen, and the meta position appeared more favorable within this limited set. Compounds **6c**, **6e**, and **6f** showed higher activity than several other analogs in the FaDu assay. While the data do not identify a molecular target, the observed differences could reflect substituent‐driven changes in physicochemical properties (e.g., polarity, lipophilicity) that affect cellular uptake, intracellular distribution, or general interaction propensity. Overall, these observations provide preliminary SAR signals that can guide future optimization.

**Figure 5:**
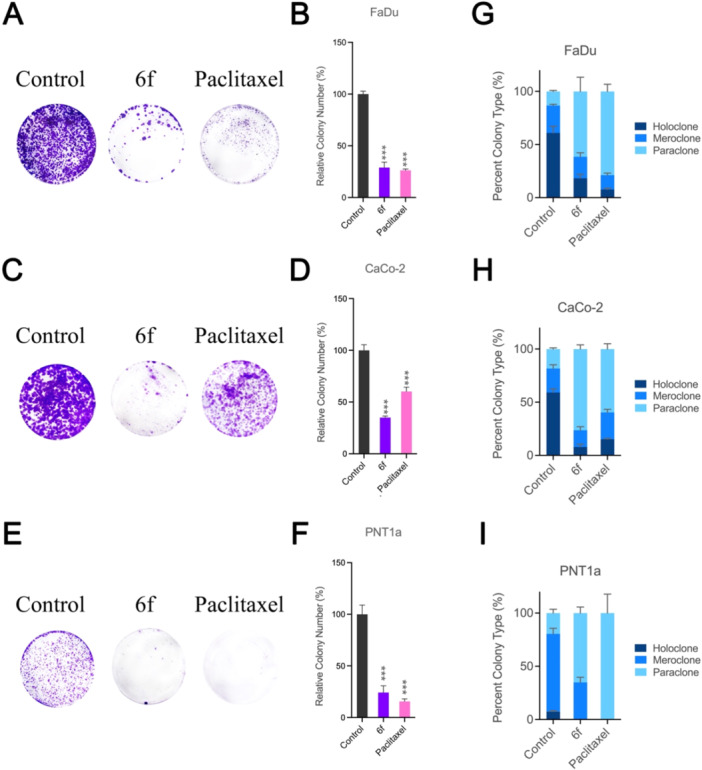
(A) Representative images of colonies formed by FaDu cells treated with **6f** or paclitaxel. (B) Relative colony number formed by FaDu cells treated with **6f** or paclitaxel as compared to controls. (C) Representative images of colonies formed by CaCo‐2 cells treated with **6f** or paclitaxel. (D) Relative colony number formed by CaCo‐2 cells treated with **6f** or paclitaxel as compared to controls. (E) Representative images of colonies formed by PNT1a cells treated with **6f** or paclitaxel. (F) Relative colony number formed by PNT1a cells treated with **6f** or paclitaxel as compared to controls. Percent colony types formed by (G) FaDu, (H) CaCo‐2, and (I) PNT1a cells treated with **6f** or paclitaxel as compared to controls. *p < 0.05, **p < 0.01, ***p < 0.001; one‐way ANOVA with Dunnett's multiple comparisons test versus control.

Meanwhile, **6f**, with relatively lower IC_50_ values in the tested cancer cell lines than in the normal epithelial PNT1a line, was selected for additional phenotypic evaluation. We investigated the effects of **6f** on single‐cell colony formation, a marker of clonogenic potential. Our results showed that **6f** reduced colony numbers in FaDu and CaCo‐2 cells compared with controls (Figure 5A–D). In PNT1a cells, **6f** also reduced colony formation, although the magnitude of the effect appeared lower than paclitaxel under the tested conditions. We emphasize that these findings do not establish a safety profile and should be interpreted as preliminary comparative observations *in vitro*. In cancer cells, the proportion of holoclones decreased, and paraclones increased after **6f** treatment (Figure 5G,H), whereas colony‐type distributions in PNT1a were less affected than with paclitaxel (Figure 5I).

**Figure 6:**
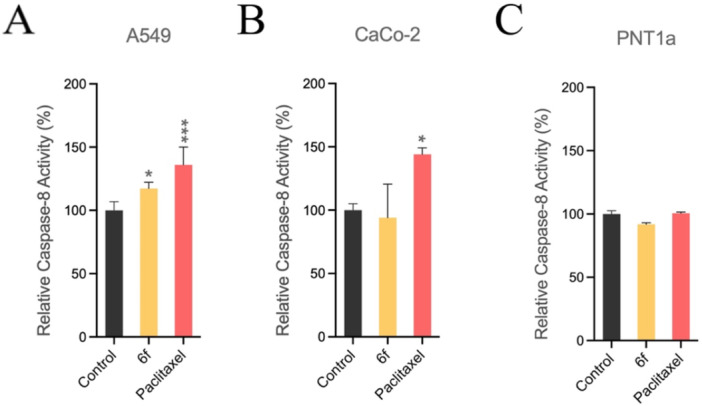
Relative Caspase‐8 activities in (A) A549, (B) CaCo‐2, and (C) PNT1a cells treated with **6f** or paclitaxel as compared to controls. *p < 0.05, ***p < 0.001; one‐way ANOVA with Dunnett's multiple comparisons test versus control.

Then, to explore whether apoptosis‐related signaling might contribute to the cellular effects of **6f**, we measured Caspase‐8 activity after treatment. We observed a significant increase in Caspase‐8 activity in A549 cells similar to paclitaxel, whereas Caspase‐8 activity was not significantly changed in CaCo‐2 and PNT1a cells under the tested conditions (Figure 6A–C). These results suggest possible involvement of extrinsic apoptosis‐associated pathways, but additional orthogonal assays (e.g., Annexin V/PI staining, caspase‐3/7 activity, PARP cleavage) would be required to draw definitive conclusions.

## 3 Conclusions

In this study, we synthesized some new dimethyl 1,2,3‐triazolesdicarboxylate‐linked (fluorine and methoxy) substituted chalcones with potential anti‐cancer activity from substituted azide chalcones and acetylene dicarboxylate by click reaction. Our findings suggest **6c**, **6e**, and especially **6f** candidate starting points for further medicinal‐chemistry optimization, although additional modifications and broader biological validation will be needed to develop more effective agents with lower IC_50_ values and to substantiate selectivity across additional normal cell models.

The SAR summary indicates that mono‐substituted derivatives exhibit higher activity than unsubstituted or multi‐methoxy substituted analogs. Among positional isomers, meta‐substituted compounds (**6c** and **6f**) demonstrated superior antiproliferative activity compared with ortho‐ or para‐substituted derivatives. In particular, the meta‐methoxy derivative **6f** showed the lowest IC_50_ values across cancer cell lines, suggesting that both electronic effects and substituent position significantly influence biological activity (Table 5).

**Table 5:** Summary of structure–activity relationship (SAR) showing the effect of substituent type and position on cytotoxic activity (IC_50_) in FaDu, A549, and CaCo‐2 cells.

Compound	Substituent	Position	FaDu IC_50_ (*µ*M)	A549 IC_50_ (*µ*M)	CaCo‐2 IC_50_ (*µ*M)	Activity note
**6a**	H	—	> 100	—	—	Inactive
**6b**	F	*ortho*	> 100	—	—	Weak
**6c**	F	*meta*	42.05	30.38	39.86	Active
**6d**	F	*para*	> 100	—	—	Weak
**6e**	OCH_3_	*ortho*	79.88	41.05	47.09	Middle
**6f**	OCH_3_	*meta*	41.12	11.46	19.88	Most active
**6g**	OCH_3_	*para*	> 100	—	—	Weak
**6h**	2,4‐diOCH_3_	mixed	> 100	—	—	Inactive
**6i**	2,3,4‐triOCH_3_	mixed	> 100	—	—	Inactive

Limitations and future directions: This study reports in vitro phenotypic screening results using a fixed concentration set and a limited panel of cell lines. Accordingly, IC_50_ estimates are interpreted conservatively, and selectivity conclusions are preliminary because only one immortalized normal epithelial line (PNT1a) was included. In addition, the triazole‐dicarboxylate products are diesters and may be susceptible to hydrolysis in vivo; evaluation of the corresponding diacid analogs, together with stability and cellular‐uptake profiling, will be important in future work. Besides, comparative testing of key intermediates (**4a**–**i**) alongside final compounds could further refine structure–activity relationships, and additional orthogonal apoptosis assays (e.g., Annexin V/PI, caspase‐3/7, PARP cleavage) would strengthen mechanistic interpretation. Finally, the relatively high variability in IC_50_ values for certain test conditions represents a limitation of this study and may be due to biological variability as well as the sensitivity of dose–response modeling. Therefore, IC_50_ values should be interpreted alongside the overall dose‐dependent trends.

## 4 Experimental

### 4.1 Chemistry

#### 4.1.1 Synthesis of 1‐(4‐azidophenyl)ethan‐1‐one (**2**)

1‐(4‐Azidophenyl)ethan‐1‐one (**2**) was synthesized according to previously reported procedures [18, 19]. To an ice‐cooled solution of **2** (1 mmol), HCl (6 M, 10 ml) was added, and the mixture was maintained at 0°C–5°C. Subsequently, an aqueous solution of sodium nitrite (1.5 mmol in 25 ml of water) was added dropwise over 30 min. Afterward, an aqueous solution of sodium azide (4 mmol in 50 ml of water) was slowly introduced, and the reaction mixture was stirred at room temperature for 24 h. Upon completion, the mixture was extracted with ethyl acetate. The combined organic layers were dried over anhydrous sodium sulfate, and the solvent was removed under reduced vacuum. After the reaction was completed, the mixture was extracted with ethyl acetate (3 × 15 ml). The crude product was used directly in the next step without further purification. Since compound 2 was employed solely as an intermediate in subsequent reactions and is well documented in the literature, its structure was characterized solely by FT‐IR spectroscopy (Scheme 1).

#### 4.1.2 General Synthesis of (*E*)−1‐(4‐azidophenyl)−3‐(substitutedphenyl)prop‐2‐en‐1‐one (**4a–i**)

The synthesis of 1‐(4‐azidophenyl)‐substituted chalcones (**4a**–**i**) was carried out via the Claisen‐Schmidt condensation method. A solution of sodium hydroxide (3 mmol) in EtOH (2.5 mL) was prepared at room temperature, to which 1‐(4‐azidophenyl)ethan‐1‐one (**2**) (1 mmol) was added. Subsequently, the corresponding substituted benzaldehyde (1.2 mmol) (**3a**–**i**) was introduced into the reaction mixture. The reaction was stirred at room temperature for 24 h. Upon completion, the reaction was poured into ice‐cold water, and the precipitated solid was collected by filtration, washed with petroleum ether, dried, and recrystallized from petroleum ether. The resulting (*E*)−1‐(4‐azidophenyl)−3‐(substitutedphenyl)prop‐2‐en‐1‐one (**4a**–**i**) were characterized by FT‐IR spectroscopy (Scheme 1) [18, 20, 21, 22].

#### 4.1.3 General Synthesis of Dimethyl (*E*)−1‐(4‐(3‐(substitutedphenyl)acryloyl)phenyl)−1*H*−1,2,3‐triazole‐4,5‐dicarboxylate (**6a‐i**)

The synthesis of dimethyl (*E*)−1‐(4‐(3‐(substitutedphenyl)acryloyl)phenyl)−1*H*−1,2,3‐triazole‐4,5‐dicarboxylate (**6a**–**i**) was accomplished via a [3 + 2] cycloaddition reaction, as previously reported. (1‐(4‐azidophenyl)chalcone (**4a**–**i**) (12 mmol) and dimethyl acetylene dicarboxylate (DMAD) **(5)** (12 mmol) were dissolved in CCl_4_ (10 ml) and refluxed. The progress of the reaction was monitored by thin‐layer chromatography (TLC). Upon completion, the solvent was removed under reduced pressure, and the crude product was purified by column chromatography using silica gel, using a solvent system of 40%–60% ethyl acetate/hexane. The product was further crystallized from DCM/hexane. The structures of compounds **6a**–**i** were confirmed by FTIR, ^1^H‐; ^13^C‐NMR, and HRMS analyses (Scheme 1).

Dimethyl (*E*)−1‐(4‐cinnamoylphenyl)−1*H*−1,2,3‐triazole‐4,5‐dicarboxylate (**6a**): The crude product was purified by column chromatography using silica gel, using a solvent system of 40%–60% ethyl acetate/hexane; 57% yield; mp.177°C–178°C; FTIR‐ATR (cm^−1^): 1721, 1663, 1608.^1^H NMR (300 MHz, CDCl_3_): *δ* 8.19 (d, *J* = 8.6 Hz, 2H, Ar‐H), 7.87 (d, *J* = 15.7 Hz, 1H, COCH═CH), 7.71 (d, *J* = 8.6 Hz, 2H, Ar‐H), 7.67 (dd, *J* = 6.5, 3.0 Hz, 2H, Ar‐H), 7.52 (d, *J* = 15.7 Hz, 1H, COCH = CH), 7.47–7.42 (m, 3H, Ar‐H), 4.02 (s, 3H, OCH_3_), 3.95 (s, 3H, OCH_3_).^13^C‐APT NMR (75 MHz, CDCl_3_): *δ* 188.9 (C═O_keto_),160.0 (O═C–O_ester_), 159.2 (O═C–O_ester_), 146.2, 139.7, 139.1(–C_triazol_), 138.4 (–C_triazol_), 134.5, 132.3, 131.0, 129.8 (2 × C), 129.0 (2 × C), 128.6 (2 × C), 124.3 (2 × C), 121.3, 54.0, 52.8. HRMS (ESI‐TOF) *m*/*z*: [M + H]^+^calculated C_21_H_17_N_3_O_5_^+^: 392.1241; found: 392.1245.

Dimethyl (*E*)−1‐{4‐[3‐(2‐fluorophenyl)acryloyl]phenyl}−1*H*−1,2,3‐triazole‐4,5‐dicarboxylate (**6b**): The crude product was purified by column chromatography using silica gel, using a solvent system of 40%–60% ethyl acetate/hexane; 65% yield; mp. 158°C; FTIR‐ATR (cm^−1^): 1721, 1660, 1606. ^1^H NMR (500 MHz, CDCl_3_): *δ* 8.20 (d, *J* = 8.6 Hz, 2H, Ar‐H), 7.95 (d, *J* = 15.9 Hz, 1H, COCH═CH), 7.72 (d, *J* = 8.6 Hz, 2H, Ar‐H), 7.66 (d, *J* = 13.8 Hz, 1H, Ar‐H), 7.65 (d, *J* = 15.8 Hz, 1H, COCH═CH), 7.42 (q, *J* = 6.5, 6.0 Hz, 1H, Ar‐H), 7.22 (t, *J* = 15.9, 8.6 Hz, 1H, Ar‐H), 7.16 (dd, *J* = 10.4, 8.7, 1H, Ar‐H), 4.02 (s, 3H, OCH_3_), 3.95 (s, 3H, OCH_3_). ^13^C‐APT NMR (126 MHz, CDCl_3_): δ 188.9 (C═O_keto_), 161.8 (d, *J* = 254.9 Hz) (–C_Ar‐F_), 160.0 (O═C–O_ester_), 159.2 (O═C–O_ester_), 139.5 (2 × C), 139.1 (–C_triazol_), 138.9 (d, *J* = 1.8 Hz), 138.5 (–C_triazol_), 132.3 (d, *J* = 8.9 Hz), 130.0 (d, *J* = 2.7 Hz), 129.9 (2 × C), 124.6 (d, *J* = 3.6 Hz), 124.3 (2 × C), 123.8 (d, *J* = 7.7 Hz), 122.6(d, *J* = 11.7 Hz), 116.43 (d, *J* = 22.0 Hz), 54.0, 52.8. HRMS (ESI‐TOF) m/z: [M + H]^+^ calculated C_21_H_16_FN_3_O_5_^+^: 410.1146; found: 410.1148.

Dimethyl (*E*)−1{4‐[3‐(3‐fluorophenyl)acryloyl]phenyl}−1*H*−1,2,3‐triazole‐4,5‐dicarboxylate (**6c**): The crude product was purified by column chromatography using silica gel, using a solvent system of 40%–60% ethyl acetate/hexane; 47% yield; mp. 151°C; FTIR‐ATR (cm^−1^): 1723, 1667, 1610.^1^H NMR (500 MHz, CDCl_3_): *δ* 8.19 (d, *J* = 7.7 Hz, 2H, Ar‐H), 7.82 (d, *J* = 15.6 Hz, 1H, COCH═CH), 7.72 (d, *J* = 7.7 Hz, 2H, Ar‐H), 7.52 (d, *J* = 15.6 Hz, 1H, COCH = CH), 7.43 (s, 2H, Ar‐H), 7.37 (d, *J* = 9.5 Hz, 1H, Ar‐H), 7.15 (t, *J* = 7.5 Hz, 1H, Ar‐H), 4.02 (s, 3H, OCH_3_), 3.96 (s, 3H, OCH_3_).^13^C‐APT NMR (126 MHz, CDCl_3_): *δ* 188.6 (C═O_keto_), 163.0 (d, *J* = 247.3 Hz) (−C_Ar‐F_), 160.0 (O═C–O_ester_), 159.2(O═C–O_ester_), 144.6 (d, *J* = 2.7 Hz), 139.4, 139.2 (–C_triazol_), 138.6 (–C_triazol_), 136.7 (d, *J* = 7.6 Hz), 132.3, 130.6 (d, *J* = 8.2 Hz), 129.8 (2 × C), 124.7 (d, *J* = 2.8 Hz), 124.4 (2 × C), 122.4, 117.8 (d, *J* = 21.5 Hz), 114.6 (d, *J* = 21.9 Hz), 54.0, 52.8. HRMS (ESI‐TOF): m/z [M + H]^+^ calculated C_21_H_16_FN_3_O_5_^+^: 410.1146; found: 410.1167.

Dimethyl (*E*)−1‐{4‐[3‐(4‐fluorophenyl)acryloyl]phenyl}−1*H*−1,2,3‐triazole‐4,5‐dicarboxylate (**6d**): The crude product was purified by column chromatography using silica gel, using a solvent system of 40%–60% ethyl acetate/hexane; 72% yield; mp. 165°C; FTIR‐ATR (cm^−1^): 1721, 1664, 1596. ^1^H NMR (500 MHz, DMSO‐*d*_6_): δ 8.32 (d, *J* = 8.4 Hz, 2H, Ar‐H), 7.95 (dd, *J* = 8.2, 5.8 Hz, 2H, Ar‐H), 7.91 (d, *J* = 15.7 Hz, 1H, COCH═CH), 7.79 (d, *J* = 8.5 Hz, 2H, Ar‐H), 7.76 (d, *J* = 15.8 Hz, 1H, COCH═CH), 7.26 (t, *J* = 8.7 Hz, 2H, Ar‐H), 3.87 (s, 3H, OCH_3_), 3.82 (s, 3H, OCH_3_).^13^C‐APT NMR (126 MHz, DMSO‐*d*_6_): *δ* 188.6 (C═O_keto_), 164.0 (d, *J* = 249.6 Hz) (–C_Ar‐F_), 160.2 (O═C–O_ester_), 159.0 (O═C–O_ester_), 144.1, 139.4, 139.0 (–C_triazol_), 138.7 (–C_triazol_), 132.2, 131.9 (d, *J* = 8.7 Hz), 131.7 (d, *J* = 3.0 Hz), 130.4(2xC), 125.4 (2 × C), 122.1 (2 × C) (d, *J* = 2.2 Hz), 116.4 (2 × C) (d, *J* = 21.8 Hz), 54.5, 53.2. HRMS (ESI‐TOF): *m*/*z* [M + H]^+^ calculated C_21_H_16_FN_3_O_5_^+^: 410.1146; found: 410.1147.

Dimethyl (*E*)−1‐{4‐[3‐(2‐methoxyphenyl)acryloyl])phenyl)−1*H*−1,2,3‐triazole‐4,5‐dicarboxylate (**6e**): The crude product was purified by column chromatography using silica gel, using a solvent system of 40%–60% ethyl acetate/hexane; 37% yield; mp. 112°C; FTIR‐ATR (cm^−1^): 1736, 1713, 1659, 1598. ^1^H NMR (500 MHz, CDCl_3_): *δ* 8.18 (d, *J* = 8.7 Hz, 2H, Ar‐H), 8.15 (d, *J* = 16.8 Hz, 1H, COCH═CH), 7.70 (d, *J* = 8.2 Hz, 2H, Ar‐H), 7.62 (d, *J* = 16.1 Hz, 1H, COCH═CH), 7.64 (d, *J* = 10.2 Hz, 1H, Ar‐H), 7.41 (t, *J* = 7.8 Hz, 1H, Ar‐H), 7.01 (t, *J* = 7.5 Hz, 1H, Ar‐H), 6.97 (d, *J* = 8.3 Hz, 1H, Ar‐H), 4.02 (s, 3H, OCH_3_), 3.95 (s, 3H, OCH_3_), 3.94 (s, 3H, OCH_3_). ^13^C‐APT NMR (126 MHz, CDCl_3_): *δ* 189.7 (C═O_keto_), 160.0 (O═C–O_ester_), 159.3 (O═C–O_ester_), 159.0 (–C_–OCH3_), 141.9, 140.1, 139.1 (–C_triazol_), 138.2 (–C_triazol_), 132.4, 132.3, 129.8 (2 × C), 129.5, 124.2 (2 × C), 123.5, 122.2, 120.8, 111.3, 55.6, 54.0, 52.8. HRMS (ESI‐TOF): *m*/*z* [M + H]^+^ calculated C_22_H_19_N_3_O_6_^+^: 422.1346; found: 422.1350.

Dimethyl (*E*)−1‐{4‐[3‐(3‐methoxyphenyl)acryloyl]phenyl}−1*H*−1,2,3‐triazole‐4,5‐dicarboxylate (**6f**): The crude product was purified by column chromatography using silica gel, using a solvent system of 40%–60% ethyl acetate/hexane; 63% yield; mp. 150°C; FTIR‐ATR (cm^−1^): 1729, 1661, 1602. ^1^H NMR (300 MHz, CDCl_3_): *δ* 8.18 (d, *J* = 8.6 Hz, 2H, Ar‐H), 7.83 (d, *J* = 15.7 Hz, 1H, COCH═CH), 7.71 (d, *J* = 8.6 Hz, 2H, Ar‐H), 7.49 (d, *J* = 15.7 Hz, 1H, COCH═CH), 7.36 (t, *J* = 7.9 Hz, 1H, Ar‐H), 7.26 (t, *J* = 3.8 Hz, 1H, Ar‐H), 7.17 (s, 1H), 7.00 (dd, *J* = 8.1, 2.4 Hz, 1H, Ar‐H), 4.02 (s, 3H, OCH_3_), 3.95 (s, 3H, OCH_3_), 3.87 (s, 3H, OCH_3_).^13^C‐APT NMR (75 MHz, CDCl_3_): *δ* 188.9 (C═O_keto_), 160.1 (O═C–O_ester_), 160.0 (O═C–O_ester_), 159.2 (–C_–OCH3_), 146.1, 139.7, 139.1 (–C_triazol_), 138.4 (–C_triazol_), 135.9, 135.8, 130.0, 129.8 (2 × C), 124.3 (2 × C), 121.6, 121.2, 116.7, 113.6, 55.3, 54.0, 52.8. HRMS (ESI‐TOF): *m*/*z* [M + H]^+^ calculated C_22_H_19_N_3_O_6_^+^: 422.1346; found: 422.1345.

Dimethyl (*E*)−1‐{4‐[3‐(4‐methoxyphenyl)acryloyl]phenyl}−1*H*−1,2,3‐triazole‐4,5‐dicarboxylate (**6g**): The crude product was purified by column chromatography using silica gel, using a solvent system of 40%–60% ethyl acetate/hexane; 40% yield; mp. 144°C; FTIR‐ATR (cm^−1^): 1732, 1647, 1600. ^1^H NMR (300 MHz, CDCl_3_): *δ* 8.18 (d, *J* = 8.5 Hz, 2H, Ar‐H), 7.84 (d, *J* = 15.6 Hz, 1H, COCH═CH), 7.70 (d, *J* = 8.5 Hz, 2H, Ar‐H), 7.63 (d, *J* = 8.8 Hz, 2H, Ar‐H), 7.40 (d, *J* = 15.6 Hz, 1H, COCH═CH), 6.96 (d, *J* = 8.7 Hz,2H, Ar‐H), 4.02 (s, 3H, OCH_3_), 3.95 (s, 3H, OCH_3_), 3.87 (s, 3H, OCH_3_).^13^C‐APT NMR (75 MHz, CDCl_3_): *δ* 188.8 (C═O_keto_), 162.0 (O═C–O_ester_), 160.0 (O═C–O_ester_), 159.2 (–C_–OCH3_), 146.0, 140.0, 139.0 (–C_triazol_), 138.2 (–C_triazol_), 132.3, 130.5 (2 × C), 129.7 (2 × C), 127.1, 124.2 (2 × C), 118.9, 114.5 (2 × C), 55.4, 54.0, 52.7. HRMS (ESI‐TOF): *m*/*z* [M + H]^+^ calculated C_22_H_19_N_3_O_6_^+^: 422.1346; found: 422.1346.

Dimethyl (*E*)−1‐{4‐[3‐(2,4‐dimethoxyphenyl)acryloyl]phenyl}−1*H*−1,2,3‐triazole‐4,5‐dicarboxylate (**6h**): The crude product was purified by column chromatography using silica gel, using a solvent system of 40%–60% ethyl acetate/hexane; 58% yield; mp. 125°C; FTIR‐ATR (cm^−1^): 1733, 1653, 1601. ^1^H NMR (500 MHz, CDCl_3_): *δ* 8.16 (d, *J* = 8.3 Hz, 2H, Ar‐H), 8.08 (d, *J* = 15.7 Hz, 1H, COCH═CH), 7.68 (d, *J* = 8.3 Hz, 2H, Ar‐H), 7.58 (d, *J* = 8.6 Hz,1H, Ar‐H), 7.53 (d, *J* = 15.7 Hz, 1H, COCH═CH), 6.55 (d, *J* = 8.6 Hz,1H, Ar‐H), 6.51–6.46 (m, 1H, Ar‐H), 4.01 (s, 3H, OCH_3_), 3.95 (s, 3H, OCH_3_), 3.92 (s, 3H, OCH_3_), 3.87 (s, 3H, OCH_3_). ^13^C‐APT NMR (126 MHz, CDCl_3_): *δ* 189.68 (C═O_keto_), 163.5 (O═C–O_ester_), 160.6 (O═C–O_ester_), 160.0 (–C_–OCH3_), 159.3 (–C_–OCH3_), 142.0, 140.5, 139.0 (–C_triazol_), 138.0, 132.4 (–C_triazol_), 131.4, 129.7 (2 × C), 124.1 (2 × C), 119.6, 116.7, 105.6, 98.5, 55.6, 55.5, 54.0, 52.8. HRMS (ESI‐TOF): *m*/*z* [M + H]^+^ calculated C_23_H_21_N_3_O_7_^+^: 452,1457; found: 452,1485.

Dimethyl (*E*)−1‐{4‐[3‐(2,3,4‐trimethoxyphenyl)acryloyl]phenyl}−1*H*−1,2,3‐triazole‐4,5 dicarboxylate (**6i**): The crude product was purified by column chromatography using silica gel, using a solvent system of 40%–60% ethyl acetate/hexane; 64% yield; mp. 130°C; FTIR‐ATR (cm^−1^): 1752, 1723, 1654, 1564. ^1^H NMR (500 MHz, CDCl_3_): *δ* 8.18 (d, *J* = 8.2 Hz, 2H, Ar‐H), 8.04 (d, *J* = 15.8 Hz, 1H, COCH═CH), 7.70 (d, *J* = 8.1 Hz, 2H, Ar‐H), 7.55 (d, *J* = 15.8 Hz, 1H, COCH═CH), 7.40 (d, *J* = 8.7 Hz, 1H, Ar‐H), 6.74 (d, *J* = 8.8 Hz, 1H, Ar‐H), 4.02 (s, 3H, OCH_3_), 3.97 (s, 3H, OCH_3_), 3.95 (s, 3H, OCH_3_), 3.93 (s, 3H, OCH_3_), 3.90 (s, 3H, OCH_3_).^13^C‐APT NMR (126 MHz, CDCl_3_): *δ* 189.3 (C═O_keto_), 160.0 (O═C–O_ester_), 159.3 (O═C–O_ester_), 156.3 (–C_–OCH3_), 154.0 (–C_–OCH3_), 142.5 (–C_–OCH3_), 141.6, 140.2, 139.1 (–C_triazol_), 138.2, 132.4 (–C_triazol_), 129.8 (2 × C), 124.2 (3 × C), 121.5, 120.5, 107.6, 61.4, 60.9, 56.1, 54.0, 52.8. HRMS (ESI‐TOF): *m*/*z* [M + H]^+^calculated C_24_H_23_N_3_O_8_^+^: 482.1557; found: 482.1557.

### 4.2 Biological Assays

#### 4.2.1 Cell Lines and Cell Culture

Normal prostate epithelial PNT1a cells, FaDu head and neck squamous cell carcinoma cell line of hypopharyngeal origin, CaCo‐2 human colon cancer cell line, and A549 human lung adenocarcinoma cell line were used to evaluate cell viability after treatment with test compounds. PNT1a, FaDu, and A549 cells were maintained in 10% fetal bovine serum (Ecotech Biotechnology, Turkiye), 1 mm l‐Glutamine (Ecotech Biotechnology, Turkiye), and 100 U/ML Penicillin/Streptomycin (Ecotech Biotechnology, Turkiye) supplemented RPMI 1640 (Ecotech Biotechnology, Turkiye) cell culture medium in 5% humidified CO_2_ incubator at 37°C. CaCo‐2 cell line was cultured in DMEM High Glucose (Ecotech Biotechnology, Turkiye) containing 10% fetal bovine serum (Ecotech Biotechnology, Turkey), 1 mM l‐Glutamine (Ecotech Biotechnology, Turkiye), and 100 U/ml Penicillin/Streptomycin (Ecotech Biotechnology, Turkiye) in 5% humidified CO_2_ incubator at 37°C.

#### 4.2.2 Cell Viability Assay

The effects of the test compounds on the vitality of PNT1a, FaDu, A549, and CaCo‐2 cells were measured using the Cell Viability Detection Kit‐8 (CVDK‐8, Ecotech Biotechnology, Turkiye) according to the manufacturer's protocol. Test compounds were prepared as DMSO stock solutions and diluted into culture medium; the final DMSO concentration was kept constant across all wells and did not exceed 0.1% (v/v), and vehicle controls contained the same DMSO concentration. In brief, PNT1a cells were seeded in 96‐well plates at 3 × 10^3^ cells/well concentration. On the other hand, FaDu, A549, and CaCo‐2 cells were seeded at 2.5 × 10^3^ cells/well in 96‐well plates. After 24 h of incubation to allow cells to attach to the plates, cells were treated with test compounds (1, 5, 25, 100 µM) for 24 h. Then, CVDK‐8 reagent, 1/10 diluted in plain cell culture medium, was added to each well, and cells were incubated at 37°C for 3 h, protected from light. Color development was detected with absorbance measurement at 450 nm with Epoch 2 Microplate Spectrophotometer (BioTek, Winooski, VT, USA). The semi‐maximal inhibitory concentration (IC_50_) value for each compound was calculated using the dose‐response curves obtained via the Probit analysis Excel file.

#### 4.2.3 Single Cell Colony Assay

The effects of test compounds on the clonogenicity of cells, which implies the stemness properties of cells, were evaluated using the colony formation assay. FaDu, CaCo‐2, and PNT1a cells were seeded in six‐well plates with 5000 cells per well and incubated for 24 h to let individual cells attach to the plates. At the end of this period, cells were treated with the IC_50_ concentrations of the test compounds. Paclitaxel was used as a positive control. During the treatment, the cell culture medium was changed with medium containing the test compound every 3–4 days. The cells were incubated for approximately 2 weeks to allow colony formation. At the end of 2 weeks, colonies were fixed with NutriCulture CrystalStain, Cell Staining Solution (Ecotech Biotechnology, Turkiye) following the manufacturer's instructions. Colonies were counted using an inverted microscope and morphologically classified as previously described [17]. Round colonies consisting of tightly packed small cells were considered as holoclones, colonies consisting of larger cells in a scattered form with a spindle structure or flat appearance were evaluated as paraclones, and colonies with intermediate characteristics of holoclone and paraclone type colonies were called meroclones [19].

#### 4.2.4 Caspase‐8 Activity Assay

Apoptotic activity of compounds was evaluated using the Colorimetric Caspase‐8 Activity Assay Kit (Ecotech Biotechnology, Turkiye) according to the manufacturer's protocol. FaDu, CaCo‐2 and PNT1a cells were seeded in 6‐well plates at a concentration of 120,000 cells per well and incubated for 24 h. Then, cells were treated with IC_50_ concentrations of test compounds and paclitaxel. Cells were incubated for 48 h to reveal caspase activity. At the end of 48 h, the medium was removed from the cells, and the wells were washed with 1× PBS. Then, cells in each well were lysed in 100 μl of lysis buffer and centrifuged at 13,000 rpm at 4°C for 10 min. 50 μg of lysate from each sample was incubated with IETD‐pNA substrate for 3 h at 37°C. Colorimetric evaluation was performed in triplicates for each group. Optical density of the samples at 405 nm was measured using a microplate reader, and percent apoptotic activity was calculated relative to the corresponding control groups.

#### 4.2.5 Statistical Analysis

Data are presented as mean ± standard error of the mean (SEM). For experiments involving more than two groups (e.g., multiple treatment conditions), one‐way ANOVA followed by an appropriate post hoc multiple‐comparison test was applied (Dunnett's test for comparisons vs. the control group). A *p*‐value < 0.05 was considered statistically significant. Data visualization and statistical analyses were performed using GraphPad Prism 9.0 (GraphPad Software Inc., San Diego, CA, USA).

## Conflicts of Interest

The authors declare no conflicts of interest.

## Supporting information

Supporting File 1

Supporting File 2

## Data Availability

The data that support the findings of this study are available in the supplementary material of this article.
