# Supplementary material for: 1,2,3‐Triazole‐Linked Chalcones as Privileged Scaffolds in Anticancer Agents
Source: Arch Pharm (Weinheim). 2026 Jul 7;359(6):e70291. doi: 10.1002/ardp.70291 (PMC13339972; doi:10.1002/ardp.70291)
Supplement: Supplementary file 1 — Supporting File 1 [file ARDP-359-e70291-s001.docx]

**1,2,3-Triazole-Linked Chalcones as Privileged Scaffolds in Anticancer Agents**

Sümeyya Koldas^a,b^, Guler Yagiz Erdemir^a^, Ahsen Kilic^c,d^, Neslisah Barlak^c,d^, Omer Faruk Karatas^c,d^, and Aliye Altundas^a^*

^a^Gazi University, Faculty of Science, Department of Chemistry, 06560, Ankara, Turkiye

^b^Gazi University, Graduate School of Natural and Applied Sciences, 06560, Ankara, Turkiye

^c^Molecular Biology and Genetics Department, Erzurum Technical University, Erzurum, Turkiye

^d^Molecular Cancer Biology Laboratory, High Technology Application and Research Center, Erzurum Technical University, Erzurum, Turkiye

**TABLE OF CONTENTS**

|  | **Chapter** | **Pages** |
| --- | --- | --- |
|  | **General experimental information** | **2-3** |
|  | **Characterization data of products** | **3-9** |
|  | **Copies of FTIR, ^1^H NMR, ^13^C NMR and HRMS spectra of the compounds** | **9-28** |
|  | **References** | **29** |

**1. Experimental**

**1.1.Material Method:**

All starting compounds were obtained from brands such as Sigma, Fluka and Merck in analytical purity and were used directly. There agent grade solvents used in the purification step were purified before use. The solvents used in there action medium were provided in anilitic purity and purified when necessary. For the synthesis of the precurs or compounds 1,2,3-Triazole-4,5-Diester Linked Chalcones as Privileged Scaffolds in Anticancer Agents S.Vanaparthi,et al. and J.A.Abbas, Y. Yıldırır were followed .Thin-layer chromatography (TLC), which was carried out on aluminum foil plates covered with silica gel 60 F254 was used to track the development of the reaction. Column chromatography using Kieselgel 60 was used to purify the product. Massspectrometry (using HRMS-TOF), Infrared and ^1^H and^13^CNMR spectros copy were used to confirm the identity and purity of the produced compounds. Using TMS as the internal standard, ^1^H and^13^C NMR spectra were recorded on device operating at 500, 300, 126 and 75 MHz, respectively. The melting points of the derivatives were recorded by the STUART (SMP-30) apparatus. Fourier-transform infrared (FTIR) spectrawerescreened on Perkin-Elmer Spectrum 100 FT-IR spectrophotometer as among 4000–500 cm^-1^.

**1.2. General synthesis of (*E*)-1-(4-azidophenyl)-3-(substitutedphenyl)prop-2-en-1-one (4a-i)**

The synthesis of 1-(4-azidophenyl)-substituted chalcones **(4a-i)** was performed via the Claisen-Schmidt condensation method. A solution of sodium hydroxide (3 mmol) in EtOH (2.5 mL) was prepared at rt, to which 1-(4-azidophenyl)ethan-1-one (**2**) (1 mmol) was added. Then, the corresponding substituted benzaldehyde (1.2 mmol) (**3a-i)** was introduced into the reaction mixture. The reaction was stirred at rt for 24 hours. Upon completion, the reaction was poured into ice-cold water, and the precipitated solid was collected by filtration, washed with PE, and dried. The resulting (*E*)-1-(4-azidophenyl)-3-(substitutedphenyl)prop-2-en-1-one **(4a-i)** were characterized by FT-IR spectroscopic technic (Scheme 1) [1,3,4,5,6].

**1.3. General synthesis of dimethyl (*E*)-1-(4-(3-(substitutedphenyl)acryloyl)phenyl)-1*H*-1,2,3-triazole-4,5-dicarboxylate (6a-i)**

The synthesis of dimethyl (*E*)-1-(4-(3-(substitutedphenyl)acryloyl)phenyl)-1*H*-1,2,3-triazole-4,5-dicarboxylate **(6a-i)** was accomplished via a [3+2] cycloaddition reaction, as previously reported [2]. (1-(4-Azidophenyl)chalcone **(4a-i)** (12 mmol) and dimethyl acetylene dicarboxylate (DMAD)**(5)** (12 mmol) were dissolved in CCl_4_ (10 mL) and refluxed. The progress of the reaction was monitored by thin-layer chromatography (TLC). After the completion of the reaction, the solvent was removed under reduced pressure. The crude product was purified by column chromatography using silica gel filter in a solvent system of 60% ethyl acetate/hexane starting from 40%. They were crystallized with DCM/hexane. Structure characterizations of the **6a-i** were carried out by FTIR, ^1^H-; ^13^C-NMR, and HRMS technics (Scheme 1)

**2.1. Characterization data of products**

Table 2.1. Physical data of 1-(4-azidophenyl)-substituted chalcone derivatives

| Name and Code of Compound | IR (ATR, cm^-1^) | Physical feature | Yield(%) |
| --- | --- | --- | --- |
| (*E*)-1-(4-azidophenyl)-3-phenylprop-2-en-1-one **4a** [3,4,6] | 2097; 1650;  1595 | Brown colored solid  MP:103-104 | 81 |
| (*E*)-1-(4-azidophenyl)-3-(2-fluorophenyl)prop-2-en-1-one **4b** [3] | 2121-2092; 1658,  1593 | Brown colored solid  MP:82-83 | 69 |
| (*E*)-1-(4-azidophenyl)-3-(3-fluorophenyl)prop-2-en-1-one **4c** [3] | 2109-2091;1650;  1595 | Brown colored solid  MP:92-93 | 75 |
| (*E*)-1-(4-azidophenyl)-3-(4-fluorophenyl)prop-2-en-1-one **4d** [3,4,6] | 2923; 2014; 1651;  1595 | Brown colored solid  MP:127 | 78 |
| (*E*)-1-(4-azidophenyl)-3-(2-methoxyphenyl)prop-2-en-1-one **4e** [5,6] | 2936-2093; 1656; 1651; 1593 | Brown colored solid  MP:150 | 85 |
| (*E*)-1-(4-azidophenyl)-3-(3-methoxyphenyl)prop-2-en-1-one **4f** | 2951; 2115-2089; 1655; 1593 | Brown colored solid  MP:79 | 80 |
| (*E*)-1-(4-azidophenyl)-3-(4-methoxyphenyl)prop-2-en-1-one **4g** [4,6] | 2966; 2110-2089; 1652; 1598 | Brown colored solid  MP:91-92 | 90 |
| (*E*)-1-(4-azidophenyl)-3-(2,4-dimethoxyphenyl)prop-2-en-1-one **4h** | 2123-2094;1650;1596 | Brown colored solid  MP:134 | 92 |
| (*E*)-1-(4-azidophenyl)-3-(2,3,4-trimethoxyphenyl)prop-2-en-1-one **4i** [5] | 2109-2089;1649;1584 | Brown colored solid  MP:85 | 85 |

**(*E*)-1-(4-Azidophenyl)-3-(3-methoxyphenyl)prop-2-en-1-one (4f)**: ^1^H NMR (300 MHz, CDCl_3_): δ 8.05 (d, *J* = 8.7 Hz, 2H, Ar-H), 7.78 (d, *J* = 15.6 Hz, 1H, COCH=CH), 7.49 (d, *J* = 15.6 Hz, 1H, COCH=CH), 7.35 (t, *J* = 7.9 Hz, 1H, Ar-H), 7.25 (d, *J* = 8.4 Hz, 2H, Ar-H), 7.13 (dd, *J* = 6.6, 4.6 Hz, 2H, Ar-H), 6.98 (dd, *J* = 8.1, 1.7 Hz, 1H, Ar-H), 3.86 (s, 3H, OCH_3_).

**(*E*)-1-(4-Azidophenyl)-3-(2,4-dimethoxyphenyl)prop-2-en-1-one (4h)** ^1^H NMR (300 MHz, CDCl_3_): δ 8.06 (d, *J* = 15.9 Hz, 1H, COCH=CH), 8.04 (d, *J* = 8.8 Hz, 2H, Ar-H), 7.57 (d, *J* = 8.7 Hz, 2H, Ar-H), 7.53 (d, *J* = 15.7 Hz, 1H, COCH=CH), 7.12 (d, *J* = 8.8 Hz, 2H, Ar-H), 6.59 – 6.43 (m, 1H, Ar-H), 3.91 (s, *J* = 3.4 Hz, 3H OCH_3_), 3.87 (s, *J* = 2.8 Hz, 3H, OCH_3_).

**Dimethyl (*E*)-1-(4-cinnamoylphenyl)-1*H*-1,2,3-triazole-4,5-dicarboxylate** (**6a**): The crude product was purified by column chromatography using silica gel filter in a solvent system of 60% ethyl acetate/hexane starting from 40%; 57% yield; mp.177-178 °C; FTIR-ATR (cm^-1^): 1721, 1663, 1608. ^1^H NMR (300 MHz,CDCl_3_): δ 8.19 (d, *J*= 8.6 Hz, 2H, Ar-H), 7.87 (d, *J* = 15.7 Hz, 1H, COCH=CH), 7.71 (d, *J* = 8.6 Hz, 2H, Ar-H), 7.67 (dd, *J* = 6.5, 3.0 Hz, 2H, Ar-H), 7.52 (d, *J* = 15.7 Hz, 1H, COCH=CH), 7.47 – 7.42 (m, 3H, Ar-H), 4.02 (s, 3H, OCH_3_), 3.95 (s, 3H, OCH_3_).^13^C-APT NMR (75 MHz, CDCl_3_): δ 188.9 (C=O_keto_),160.0 (O=C-O_ester_), 159.2(O=C-O_ester_), 146.2, 139.7, 139.1(-C_triazol_), 138.4(-C_triazol_), 134.5, 132.3, 131.0, 129.8 (2xC), 129.0(2xC), 128.6 (2xC), 124.3 (2xC), 121.3, 54.0, 52.8. HRMS (ESI-TOF) m/z: [M+H]^+^calculated C_21_H_17_N_3_O_5_^+^ : 392.1241; found: 392.1245.

**Dimethyl (*E*)-1-{4-[3-(2-fluorophenyl)acryloyl]phenyl}-1*H*-1,2,3-triazole-4,5-dicarboxylate** (**6b**): The crude product was purified by column chromatography using silica gel filter in a solvent system of 60% ethyl acetate/hexane starting from 40%; 65% yield; mp. 158 °C; FTIR-ATR (cm^-1^): 1721, 1660, 1606. ^1^H NMR (500 MHz,CDCl_3_): δ 8.20 (d, *J* = 8.6 Hz, 2H, Ar-H), 7.95 (d, *J* = 15.9 Hz, 1H, COCH=CH), 7.72 (d, *J* = 8.6 Hz, 2H, Ar-H), 7.66 (d, *J* = 13.8 Hz, 1H, Ar-H), 7.65 (d, *J* = 15.8 Hz, 1H, COCH=CH), 7.42 (q, *J* = 6.5, 6.0 Hz, 1H, Ar-H), 7.22 (t, *J* = 15.9, 8.6 Hz, 1H, Ar-H), 7.16 (dd, *J* = 10.4, 8.7, 1H, Ar-H), 4.02 (s, 3H, OCH_3_), 3.95 (s, 3H, OCH_3_). ^13^C-APT NMR (126 MHz, CDCl_3_): δ 188.9 (C=O_keto_), 161.8 (d, *J* = 254.9 Hz) (-C_Ar-F_), 160.0 (O=C-O_ester_), 159.2 (O=C-O_ester_), 139.5 (2xC), 139.1 (-C_triazol_), 138.9 (d, *J* = 1.8 Hz), 138.5 (-C_triazol_), 132.3 (d, *J* = 8.9 Hz), 130.0 (d, *J* = 2.7 Hz), 129.9 (2xC), 124.6 (d, *J* = 3.6 Hz), 124.3 (2xC), 123.8 (d, *J* = 7.7 Hz), 122.6(d, *J* = 11.7 Hz), 116.43 (d, *J* = 22.0 Hz), 54.0, 52.8. HRMS (ESI-TOF) m/z: [M+H]^+^ calculated C_21_H_16_FN_3_O_5_^+^ : 410.1146; found: 410.1148.

**Dimethyl (*E*)-1{4-[3-(3-fluorophenyl)acryloyl]phenyl}-1*H*-1,2,3-triazole-4,5-dicarboxylate (6c):** The crude product was purified by column chromatography using silica gel filter in a solvent system of 60% ethyl acetate/hexane starting from 40%; 47% yield; mp. 151°C; FTIR-ATR (cm^-1^): 1723, 1667, 1610. ^1^H NMR (500 MHz,CDCl_3_): δ 8.19 (d, *J* = 7.7 Hz, 2H, Ar-H), 7.82 (d, *J* = 15.6 Hz, 1H, COCH=CH), 7.72 (d, *J* = 7.7 Hz, 2H, Ar-H), 7.52 (d, *J* = 15.6 Hz, 1H,COCH=CH), 7.43 (s, 2H, Ar-H), 7.37 (d, *J* = 9.5 Hz, 1H, Ar-H), 7.15 (t, *J* = 7.5 Hz, 1H, Ar-H), 4.02 (s, 3H, OCH_3_), 3.96 (s, 3H, OCH_3_).^13^C-APT NMR (126 MHz, CDCl_3_): δ 188.6 (C=O_keto_), 163.0 (d, *J* = 247.3 Hz) (-C_Ar-F_), 160.0(O=C-O_ester_), 159.2(O=C-O_ester_), 144.6 (d, *J* = 2.7 Hz), 139.4, 139.2(-C_triazol_), 138.6(-C_triazol_), 136.7 (d, *J* = 7.6 Hz), 132.3, 130.6 (d, *J* = 8.2 Hz), 129.8 (2xC), 124.7 (d, *J* = 2.8 Hz), 124.4 (2xC), 122.4, 117.8(d, *J* = 21.5 Hz), 114.6 (d, *J* = 21.9 Hz), 54.0, 52.8. HRMS (ESI-TOF): m/z [M+H]^+^ calculated C_21_H_16_FN_3_O_5_^+^ : 410.1146; found:410.1167.

**Dimethyl (*E*)-1-{4-[3-(4-fluorophenyl)acryloyl]phenyl}-1*H*-1,2,3-triazole-4,5-dicarboxylate** (**6d**): The crude product was purified by column chromatography using silica gel filter in a solvent system of 60% ethyl acetate/hexane starting from 40%; 72% yield; mp. 165 °C; FTIR-ATR (cm^-1^): 1721, 1664, 1596. ^1^H NMR (500 MHz, DMSO-*d*_6_): δ 8.32 (d, *J* = 8.4 Hz, 2H, Ar-H), 7.95 (dd, *J* = 8.2, 5.8 Hz, 2H, Ar-H), 7.91 (d, *J* = 15.7 Hz, 1H, COCH=CH), 7.79 (d, *J* = 8.5 Hz, 2H, Ar-H), 7.76 (d, *J* = 15.8 Hz, 1H, COCH=CH), 7.26 (t, *J* = 8.7 Hz, 2H, Ar-H), 3.87 (s, 3H, OCH_3_), 3.82 (s, 3H, OCH_3_).^13^C-APT NMR (126 MHz, DMSO-*d*_6_): δ 188.6 (C=O_keto_), 164.0 (d, *J* = 249.6 Hz) (-C_Ar-F_), 160.2 (O=C-O_ester_), 159.0 (O=C-O_ester_), 144.1, 139.4,139.0(-C_triazol_), 138.7(-C_triazol_), 132.2, 131.9 (d, *J* = 8.7 Hz), 131.7 (d, *J* = 3.0 Hz), 130.4(2xC), 125.4 (2xC), 122.1 (2xC) (d, *J* = 2.2 Hz), 116.4 (2xC) (d, *J* = 21.8 Hz), 54.5, 53.2. HRMS (ESI-TOF): m/z [M+H]^+^ calculated C_21_H_16_FN_3_O_5_^+^ : 410.1146; found: 410.1147.

**Dimethyl (*E*)-1-{4-[3-(2-methoxyphenyl)acryloyl]phenyl}-1*H*-1,2,3-triazole-4,5-dicarboxylate** (**6e**): The crude product was purified by column chromatography using silica gel filter in a solvent system of 60% ethyl acetate/hexane starting from 40%; 37% yield; mp. 112 °C; FTIR-ATR (cm^-1^): 1736, 1713, 1659, 1598. ^1^H NMR (500 MHz, CDCl_3_): δ 8.18 (d, *J* = 8.7 Hz, 2H,Ar-H), 8.15 (d, *J* = 16.8 Hz, 1H, COCH=CH), 7.70 (d, *J* = 8.2 Hz, 2H, Ar-H), 7.62 (d, *J* = 16.1 Hz, 1H, COCH=CH), 7.64 (d, *J* = 10.2 Hz, 1H, Ar-H), 7.41 (t, *J* = 7.8 Hz, 1H, Ar-H), 7.01 (t, *J* = 7.5 Hz, 1H, Ar-H), 6.97 (d, *J* = 8.3 Hz, 1H, Ar-H), 4.02 (s, 3H, OCH_3_), 3.95 (s, 3H, OCH_3_), 3.94 (s, 3H, OCH_3_). ^13^C-APT NMR (126 MHz, CDCl_3_): δ 189.7 (C=O_keto_), 160.0(O=C-O_ester_), 159.3(O=C-O_ester_), 159.0 (-C_–OCH3_), 141.9, 140.1, 139.1(-C_triazol_), 138.2(-C_triazol_), 132.4, 132.3, 129.8 (2xC), 129.5, 124.2 (2xC), 123.5, 122.2, 120.8, 111.3, 55.6, 54.0, 52.8. HRMS (ESI-TOF): m/z [M+H]^+^ calculated C_22_H_19_N_3_O_6_^+^ : 422.1346; found:422.1350.

**Dimethyl (*E*)-1-{4-[3-(3-methoxyphenyl)acryloyl]phenyl}-1*H*-1,2,3-triazole-4,5-dicarboxylate** (**6f**): The crude product was purified by column chromatography using silica gel filter in a solvent system of 60% ethyl acetate/hexane starting from 40%; 63%yield; mp. 150 °C; FTIR-ATR (cm^-1^): 1729, 1661, 1602. ^1^H NMR (300 MHz, CDCl_3_): δ 8.18 (d, *J* = 8.6 Hz, 2H, Ar-H), 7.83 (d, *J* = 15.7 Hz, 1H, COCH=CH), 7.71 (d, *J* = 8.6 Hz, 2H, Ar-H), 7.49 (d, *J* = 15.7 Hz, 1H, COCH=CH), 7.36 (t, *J* = 7.9 Hz, 1H, Ar-H), 7.26 (t, *J* = 3.8 Hz, 1H, Ar-H), 7.17 (s, 1H), 7.00 (dd, *J* = 8.1, 2.4 Hz, 1H, Ar-H), 4.02 (s, 3H, OCH_3_), 3.95 (s, 3H, OCH_3_), 3.87 (s, 3H, OCH_3_).^13^C-APT NMR (75 MHz, CDCl_3_): δ 188.9 (C=O_keto_), 160.1 (O=C-O_ester_), 160.0 (O=C-O_ester_), 159.2(-C_–OCH3_), 146.1, 139.7, 139.1(-C_triazol_),138.4(-C_triazol_), 135.9, 135.8, 130.0, 129.8 (2xC), 124.3 (2xC), 121.6, 121.2, 116.7, 113.6, 55.3, 54.0, 52.8. HRMS (ESI-TOF): m/z [M+H]^+^ calculated C_22_H_19_N_3_O_6_^+^ : 422.1346; found:422.1345.

**Dimethyl (*E*)-1-{4-[3-(4-methoxyphenyl)acryloyl]phenyl}-1*H*-1,2,3-triazole-4,5-dicarboxylate** (**6g**): The crude product was purified by column chromatography using silica gel filter in a solvent system of 60% ethyl acetate/hexane starting from 40%;40%yield;mp. 144°C; FTIR-ATR (cm^-1^): 1732, 1647, 1600. ^1^H NMR (300 MHz, CDCl_3_): δ 8.18 (d, *J* = 8.5 Hz, 2H, Ar-H), 7.84 (d, *J* = 15.6 Hz, 1H, COCH=CH), 7.70 (d, *J* = 8.5 Hz, 2H, Ar-H), 7.63 (d, *J* = 8.8 Hz, 2H, Ar-H), 7.40 (d, *J* = 15.6 Hz, 1H, COCH=CH), 6.96 (d, *J* = 8.7 Hz, 2H,Ar-H), 4.02 (s, 3H, OCH_3_), 3.95 (s, 3H, OCH_3_), 3.87 (s, 3H, OCH_3_).^13^C-APT NMR (75 MHz, CDCl_3_): δ 188.8 (C=O_keto_), 162.0 (O=C-O_ester_), 160.0 (O=C-O_ester_), 159.2 (-C_–OCH3_), 146.0, 140.0, 139.0(-C_triazol_), 138.2 (-C_triazol_), 132.3, 130.5 (2xC), 129.7 (2xC), 127.1, 124.2 (2xC), 118.9, 114.5 (2xC), 55.4, 54.0, 52.7. HRMS (ESI-TOF): m/z [M+H]^+^ calculated C_22_H_19_N_3_O_6_^+^ : 422.1346; found:422.1346.

**Dimethyl (*E*)-1-{4-[3-(2,4-dimethoxyphenyl)acryloyl]phenyl}-1*H*-1,2,3-triazole-4,5-dicarboxylate** (**6h**): The crude product was purified by column chromatography using silica gel filter in a solvent system of 60% ethyl acetate/hexane starting from 40%; 58%yield; mp. 125 °C; FTIR-ATR (cm^-1^): 1733, 1653, 1601. ^1^H NMR (500 MHz, CDCl_3_): δ 8.16 (d, *J* = 8.3 Hz, 2H, Ar-H), 8.08 (d, *J* = 15.7 Hz, 1H, COCH=CH), 7.68 (d, *J* = 8.3 Hz, 2H, Ar-H), 7.58 (d, *J* = 8.6 Hz,1H, Ar-H), 7.53 (d, *J* = 15.7 Hz, 1H, COCH=CH), 6.55 (d, *J* = 8.6 Hz,1H,Ar-H), 6.51 – 6.46 (m, 1H, Ar-H), 4.01 (s, 3H, OCH_3_), 3.95 (s, 3H, OCH_3_), 3.92 (s, 3H, OCH_3_), 3.87 (s, 3H,OCH_3_). ^13^C-APT NMR (126 MHz, CDCl_3_): δ 189.68(C=O_keto_), 163.5 (O=C-O_ester_), 160.6 (O=C-O_ester_), 160.0 (-C_–OCH3_), 159.3 (-C_–OCH3_), 142.0, 140.5, 139.0(-C_triazol_), 138.0, 132.4(-C_triazol_), 131.4, 129.7 (2xC), 124.1(2xC), 119.6, 116.7, 105.6, 98.5, 55.6, 55.5, 54.0, 52.8. HRMS (ESI-TOF): m/z [M+H]^+^ calculated C_23_H_21_N_3_O_7_^+^ : 452,1457; found:452,1485.

**Dimethyl (*E*)-1-{4-[3-(2,3,4-trimethoxyphenyl)acryloyl]phenyl}-1*H*-1,2,3-triazole-4,5 dicarboxylate (6i):** The crude product was purified by column chromatography using silica gel filter in a solvent system of 60% ethyl acetate/hexane starting from 40%; 64%yield; mp. 130°C; FTIR-ATR (cm^-1^): 1752, 1723, 1654, 1564. ^1^H NMR (500 MHz, CDCl_3_): δ 8.18 (d, *J* = 8.2 Hz, 2H, Ar-H), 8.04 (d, *J* = 15.8 Hz, 1H, COCH=CH), 7.70 (d, J = 8.1 Hz, 2H, Ar-H), 7.55 (d, *J* = 15.8 Hz, 1H,COCH=CH), 7.40 (d, *J* = 8.7 Hz, 1H, Ar-H), 6.74 (d, *J* = 8.8 Hz, 1H, Ar-H), 4.02 (s, 3H, OCH_3_), 3.97 (s, 3H,OCH_3_), 3.95 (s, 3H, OCH_3_), 3.93 (s, 3H,OCH_3_), 3.90 (s, 3H,OCH_3_).^13^C-APT NMR (126 MHz, CDCl_3_): δ 189.3 (C=O_keto_), 160.0 (O=C-O_ester_ ), 159.3(O=C-O_ester_ ), 156.3 (-C_–OCH3_), 154.0 (-C_–OCH3_), 142.5 (-C_–OCH3_), 141.6, 140.2, 139.1(-C_triazol_), 138.2, 132.4(-C_triazol_), 129.8 (2xC), 124.2 (3xC), 121.5, 120.5, 107.6, 61.4, 60.9, 56.1, 54.0, 52.8. HRMS (ESI-TOF): m/z [M+H]^+^calculated C_24_H_23_N_3_O_8_^+^: 482.1557; found:482.1557.

**3.1 Copies of FTIR, ^1^H NMR, ^13^C NMR and HRMS spectra of the compounds**


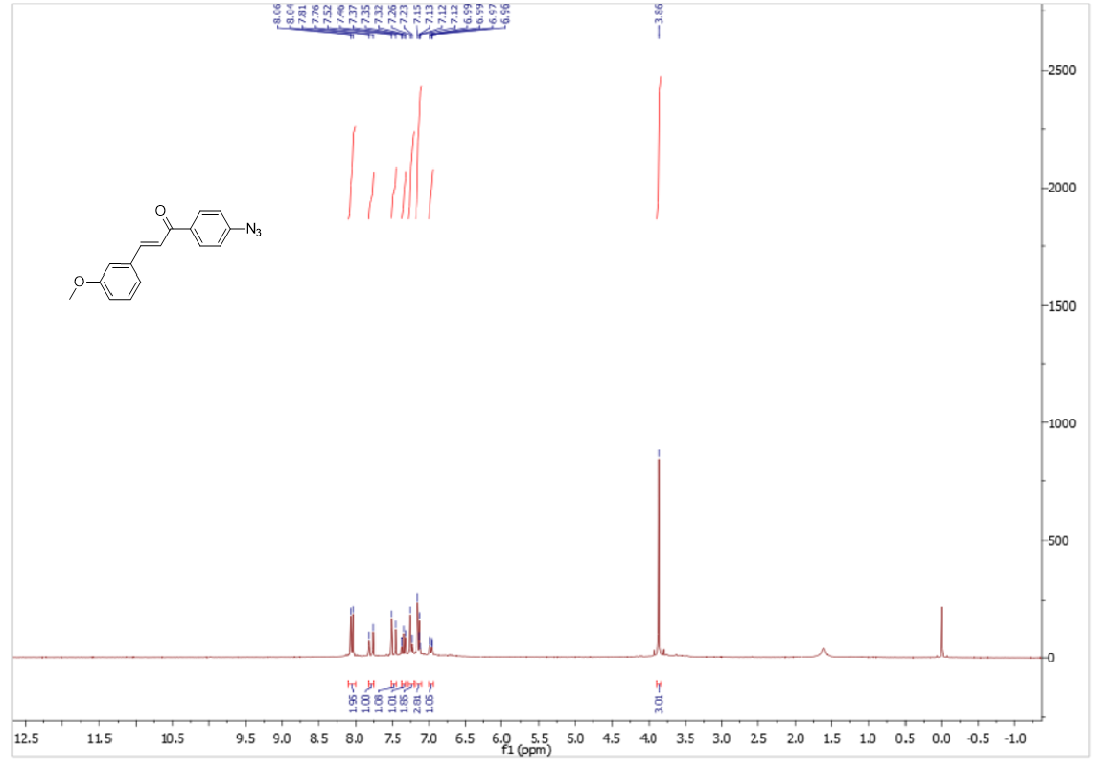


Figure S1. FTIR spectrum of Compound 4f


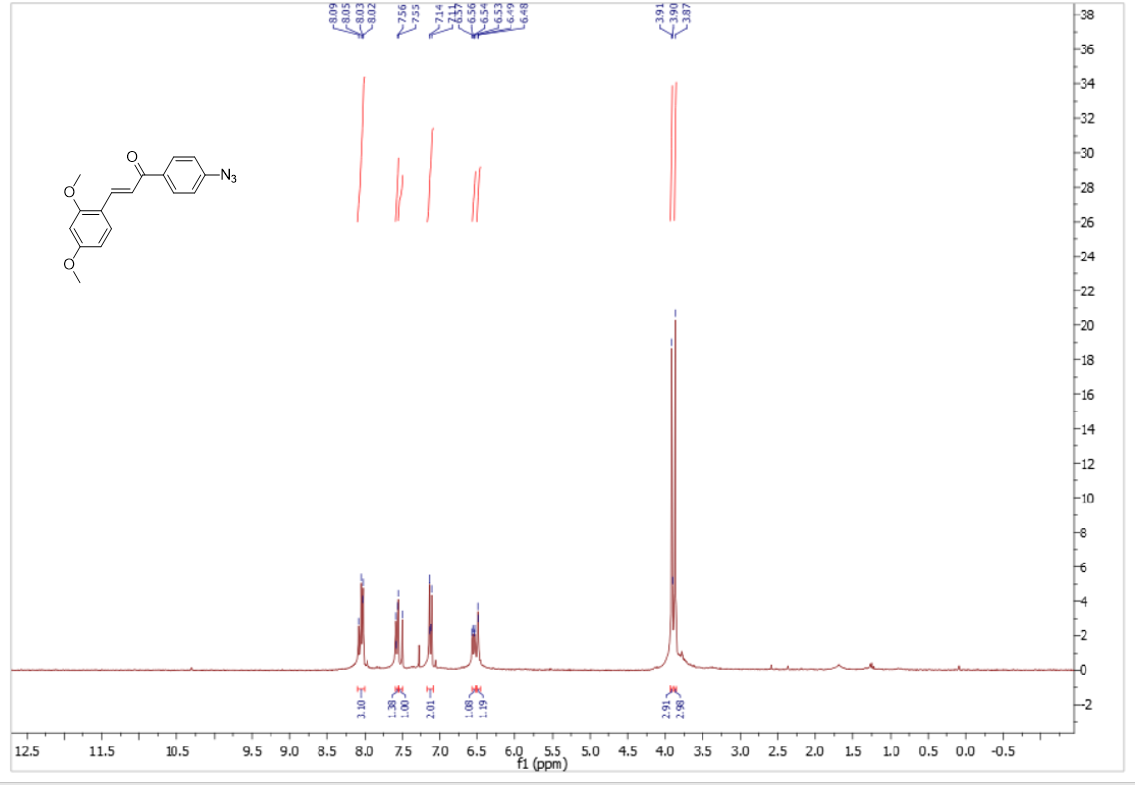


Figure S2. FTIR spectrum of Compound 4h

**
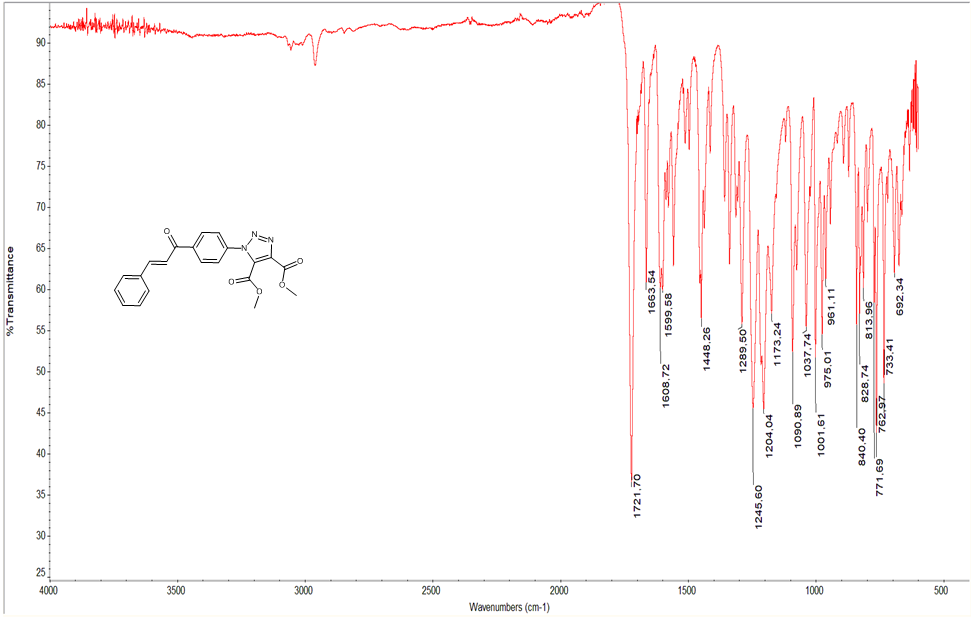
**

Figure S3. FTIR spectrum of Compound 6a


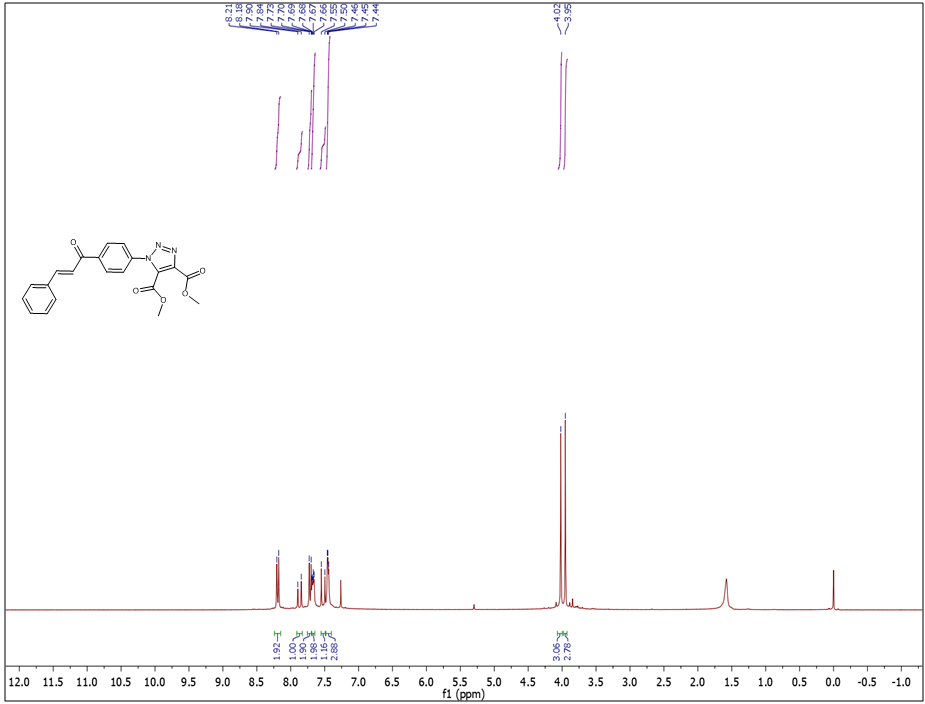


Figure S4. ^1^H NMR spectrum of Compound 6a


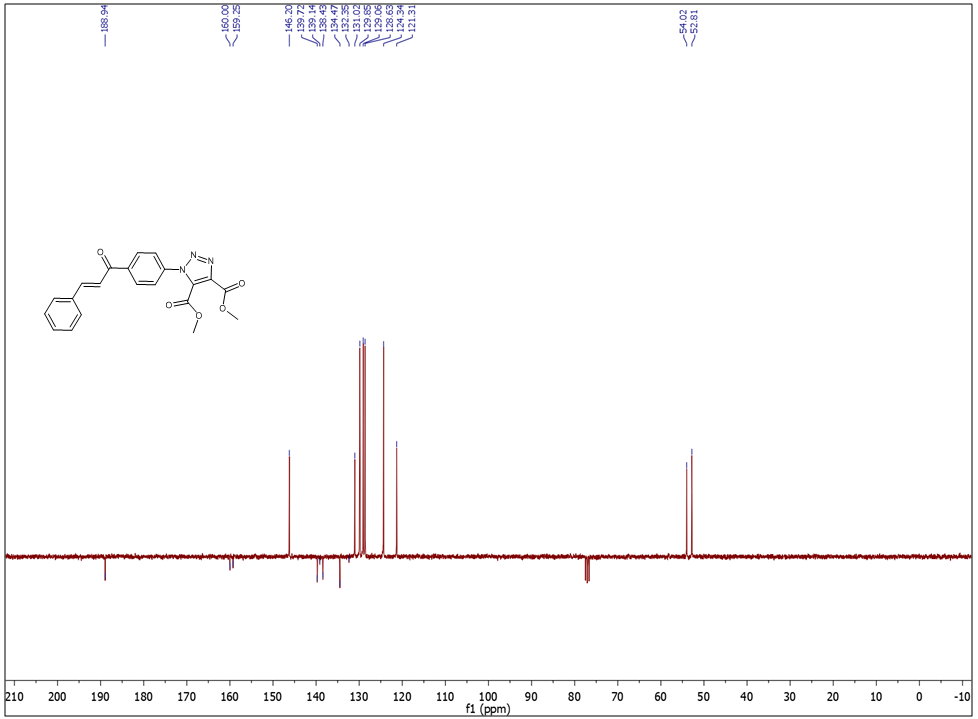


Figure S5. ^13^C-APT NMR spectrum of Compound 6a


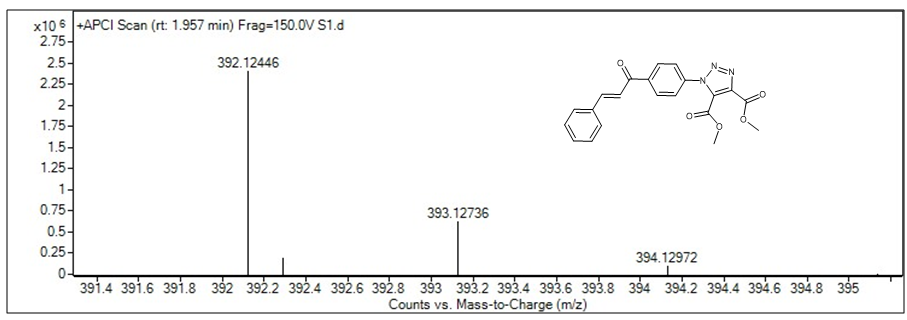


Figure S6. HRMS spectrum of Compound 6a


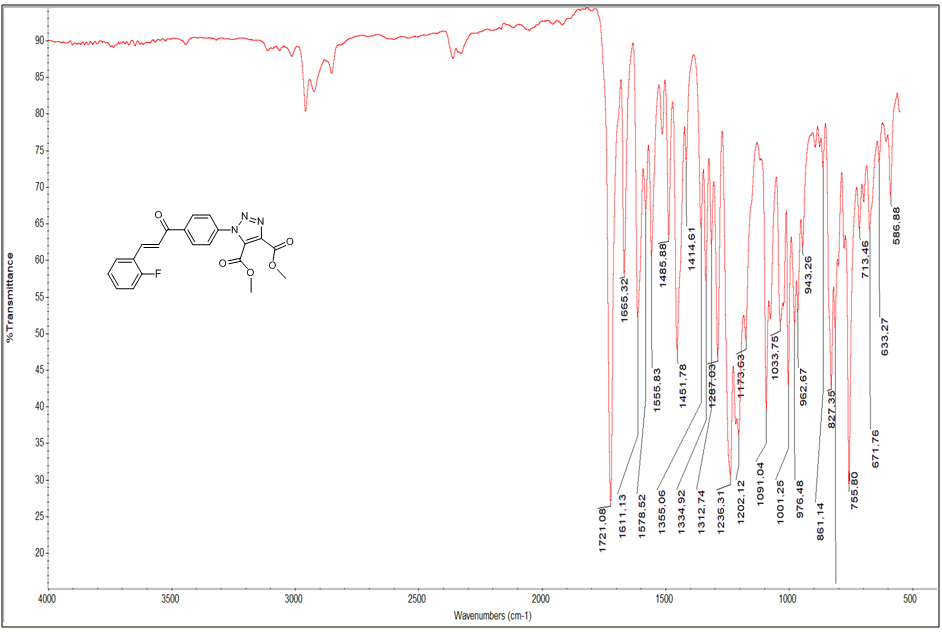


Figure S7. FTIR spectrum of Compound 6b


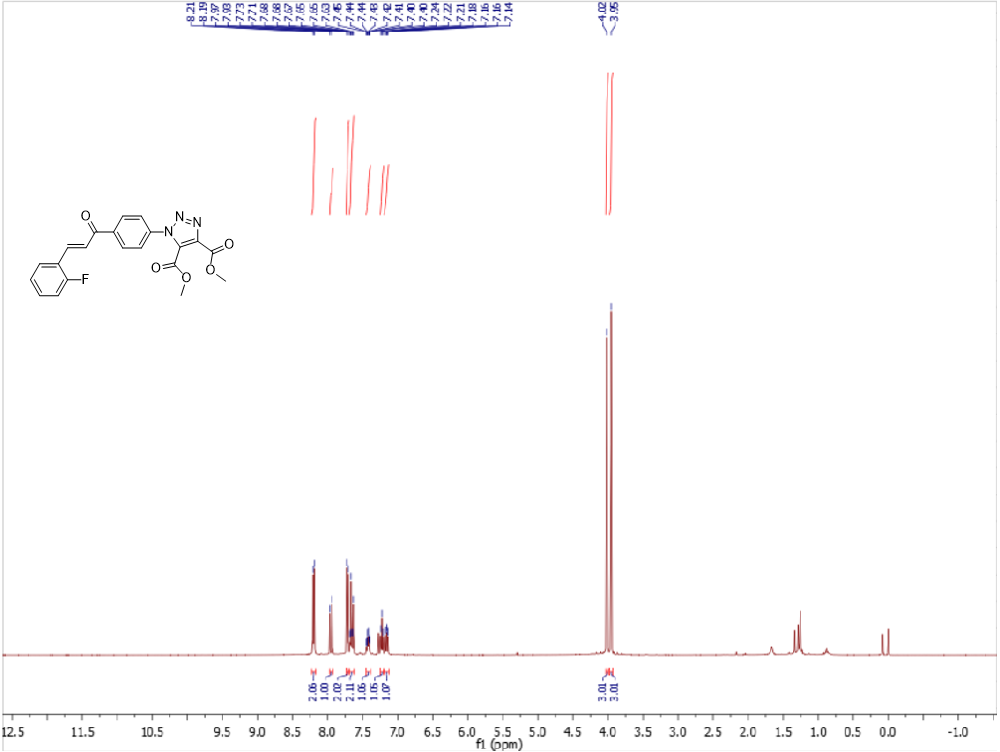


Figure S8. ^1^H NMR spectrum of Compound 6b


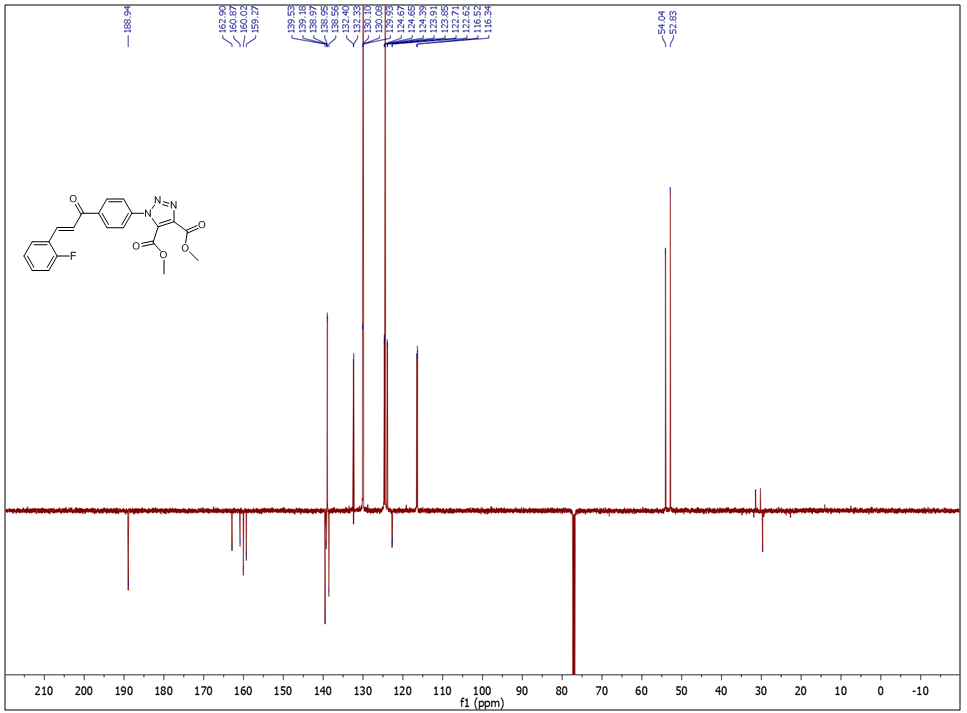
**Figure S9. ^13^C-APT NMR** spectrum of Compound **6b**


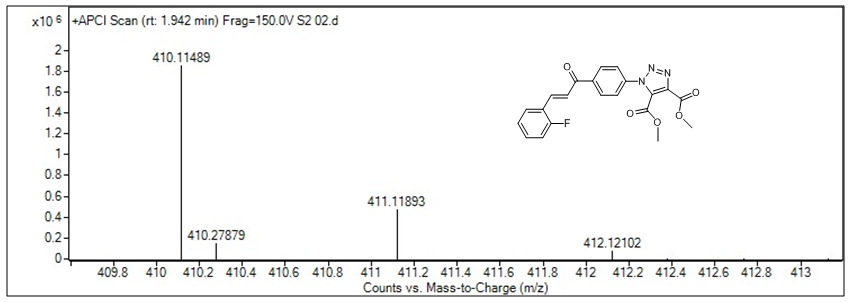


Figure S10. HRMS spectrum of Compound 6b


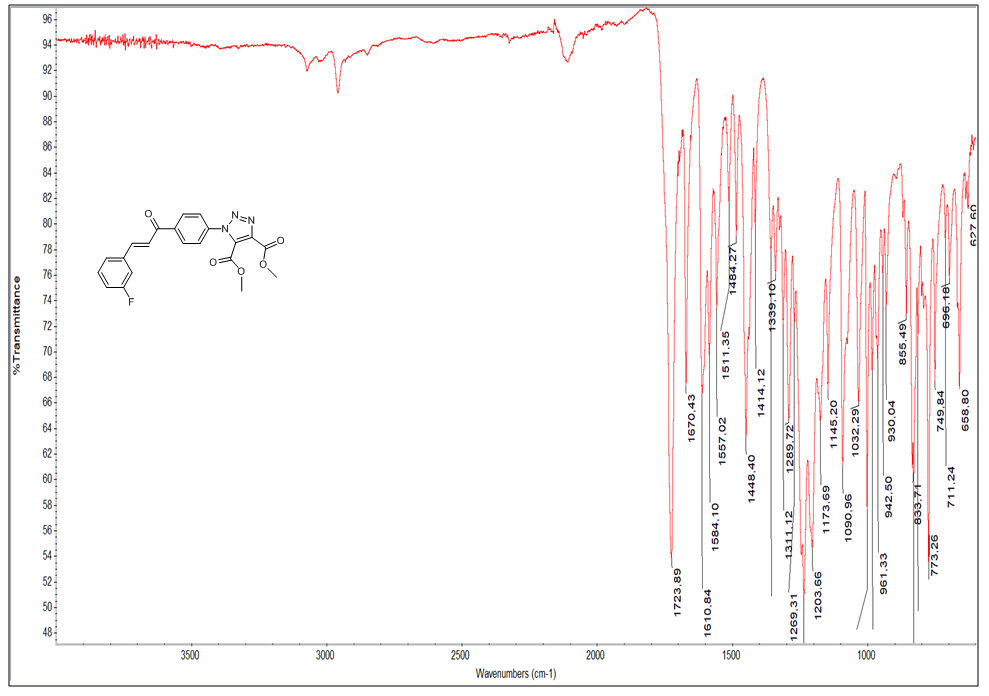


**Figure S11. FTIR** spectrum of Compound **6c**


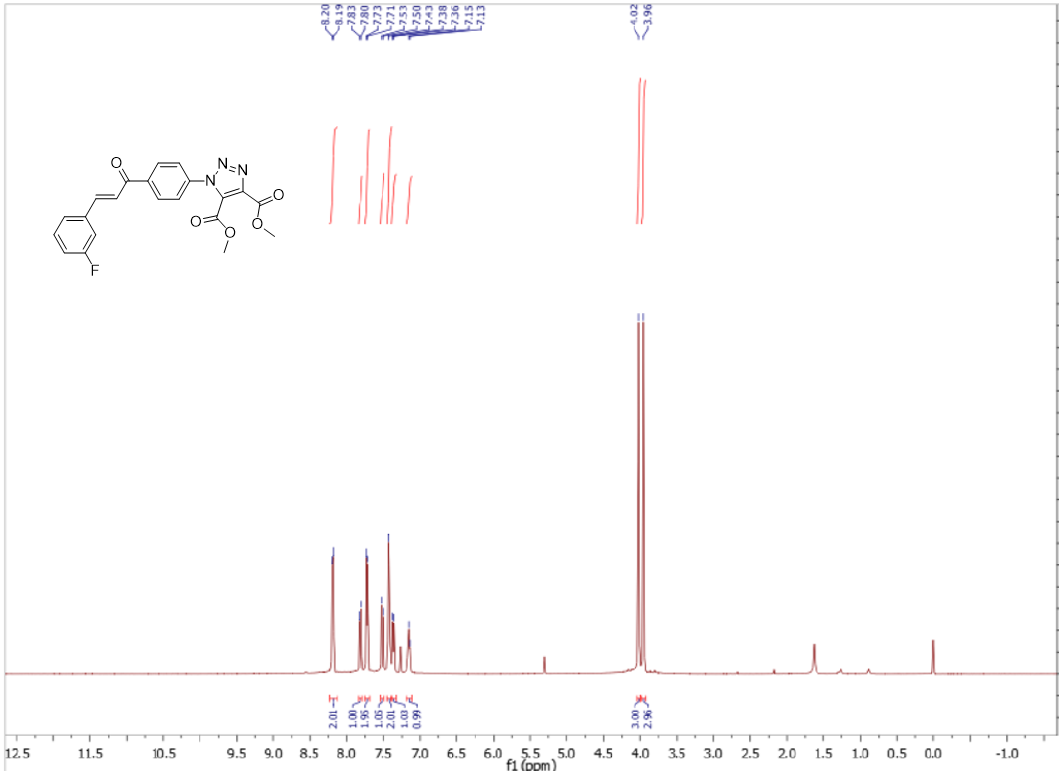


Figure S12. ^1^H NMR spectrum of Compound 6c


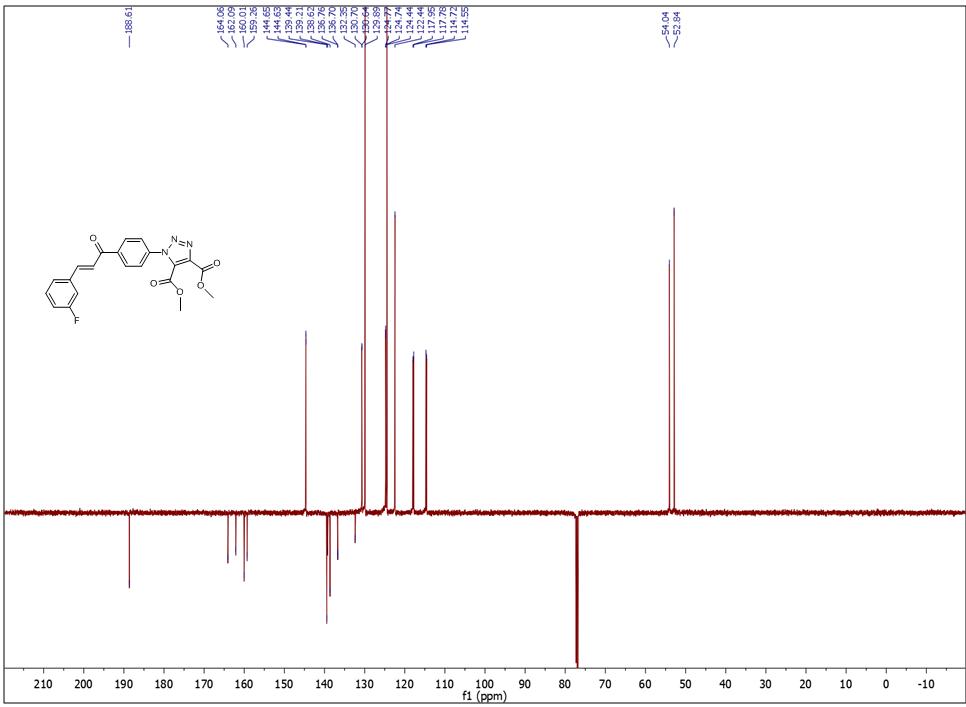
**Figure S13. ^13^C-APT NMR** spectrum of Compound **6c**


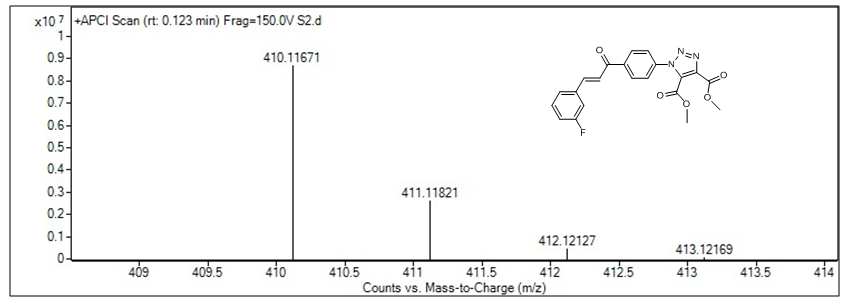


Figure S14. HRMS spectrum of Compound 6c


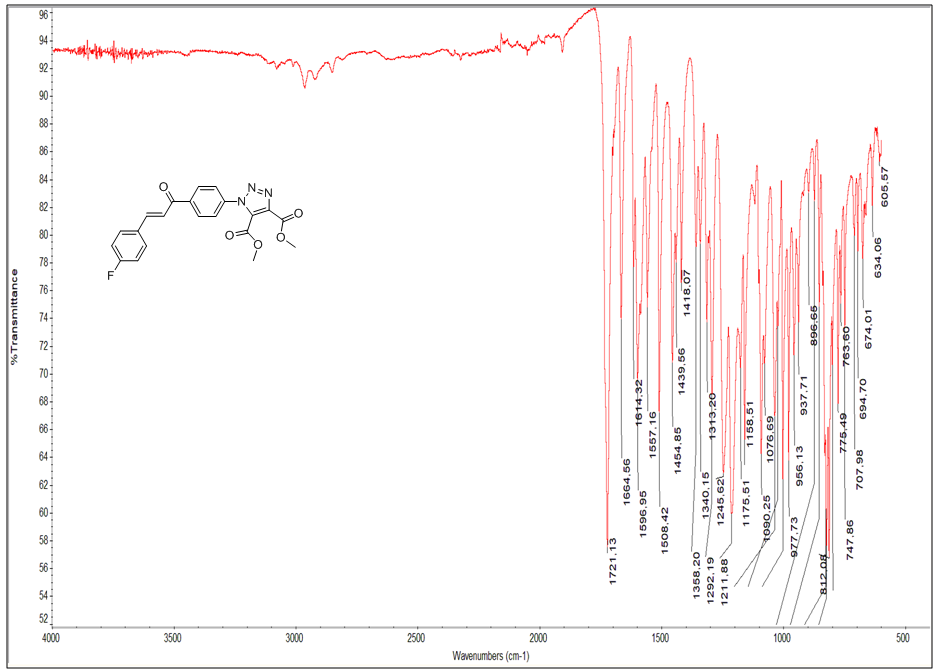


Figure S15. FTIR spectrum of Compound 6d


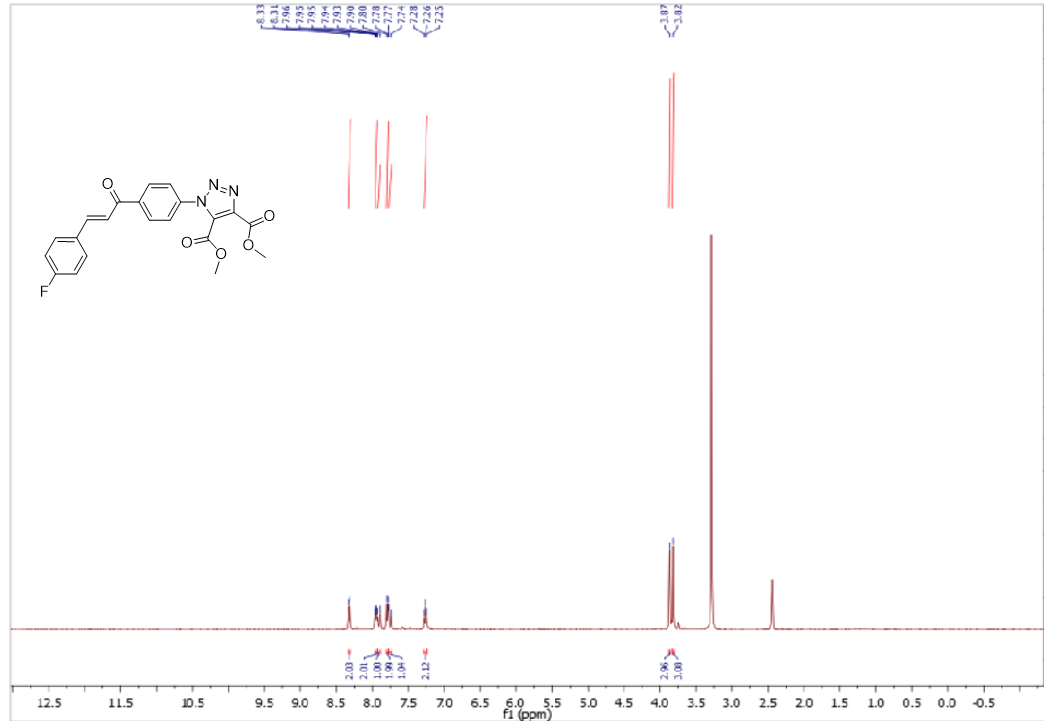


**Figure S16. ^1^H NMR** spectrum of Compound **6d**


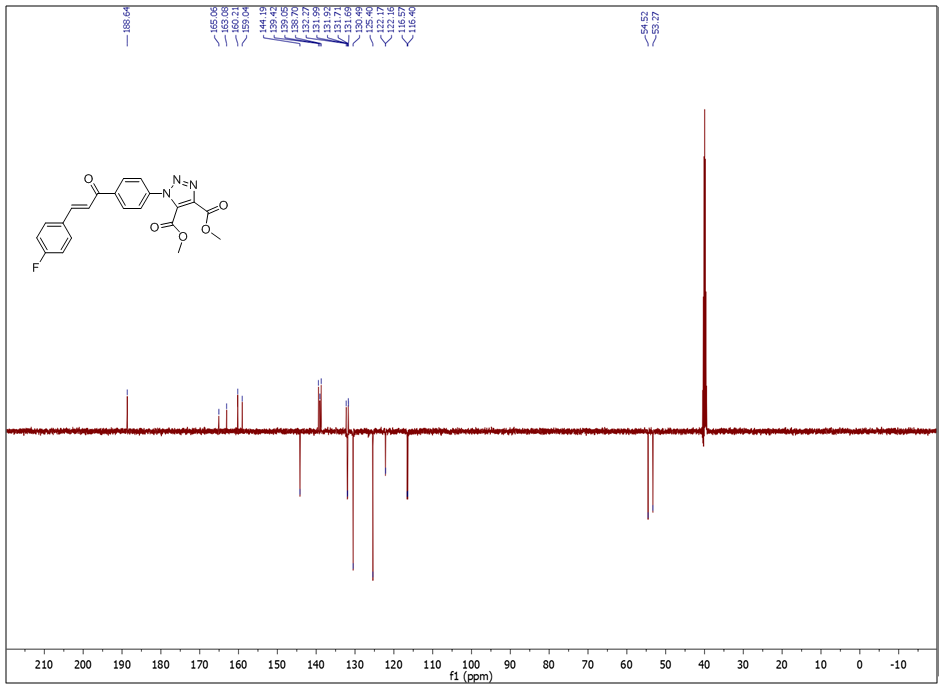


Figure S17. ^13^C-APT NMR spectrum of Compound 6d


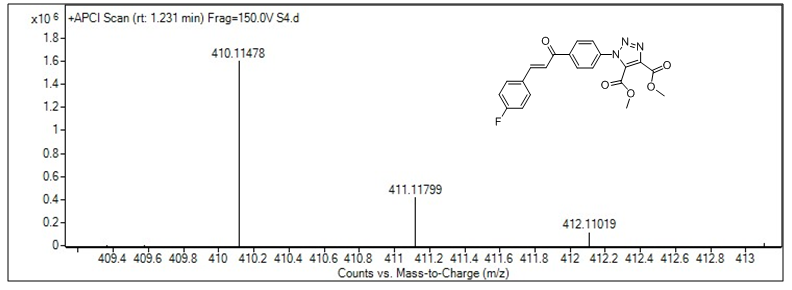


Figure S18. HRMS spectrum of Compound 6d


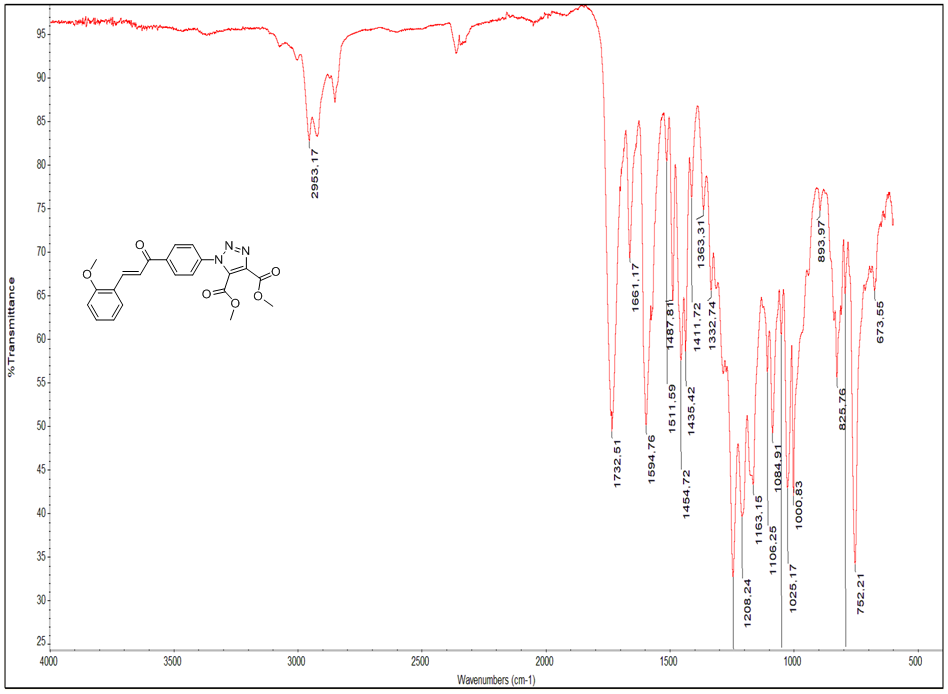


Figure S19. FTIR spectrum of Compound 6e


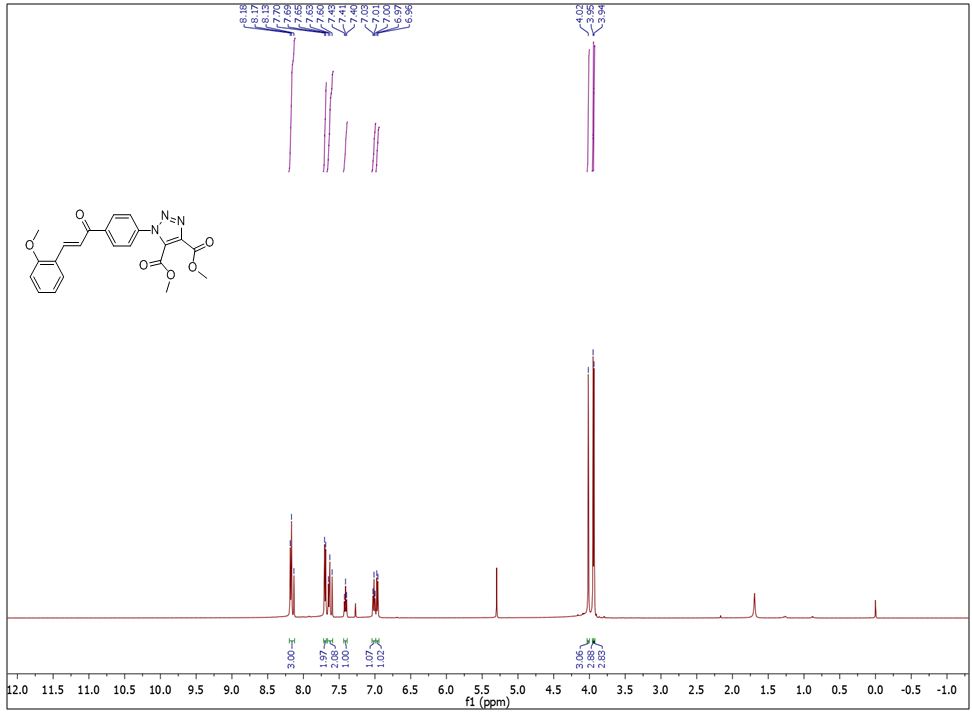


Figure S20. ^1^H NMR spectrum of Compound 6e


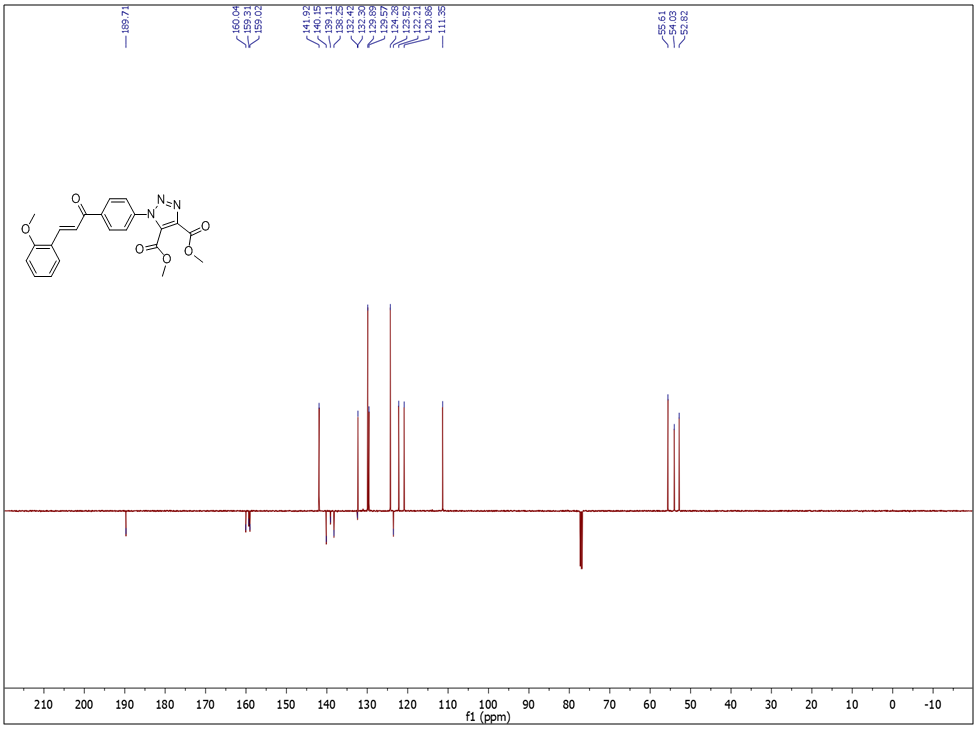


Figure S21. ^13^C-APT NMR spectrum of Compound 6e


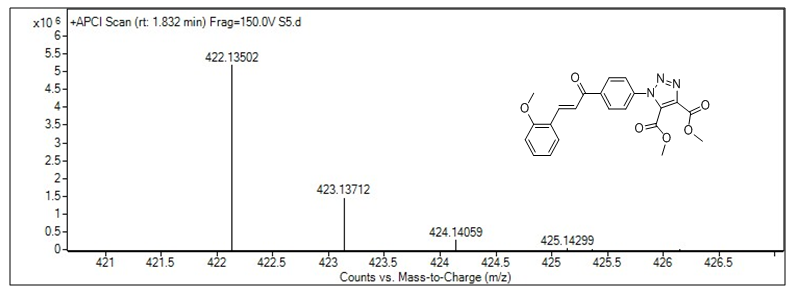


Figure S22. HRMS spectrum of Compound 6e


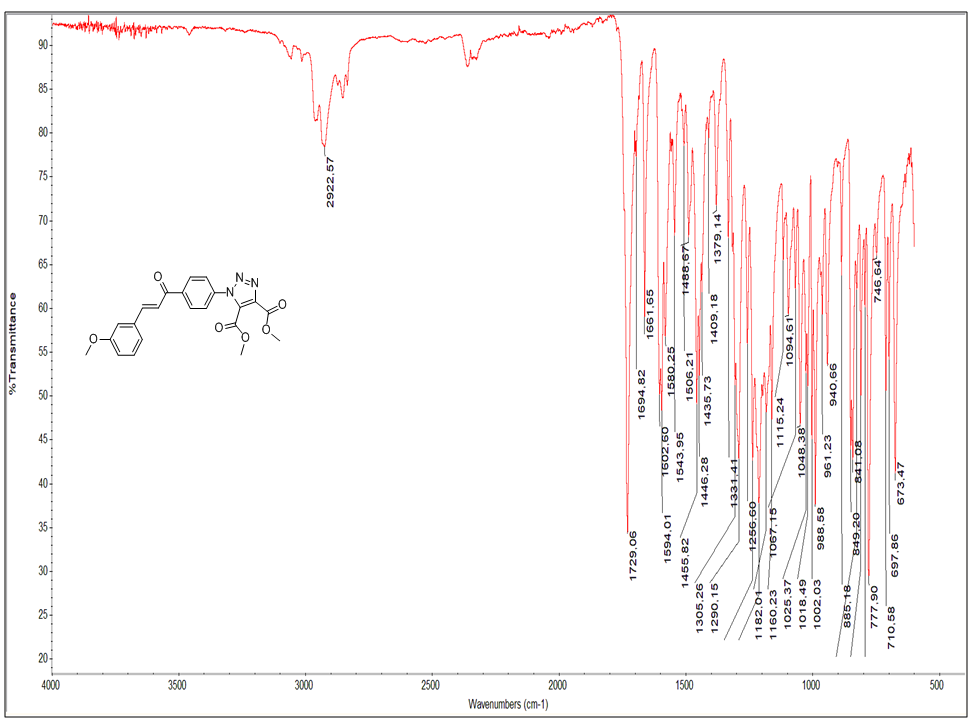


Figure S23. FTIR spectrum of Compound 6f


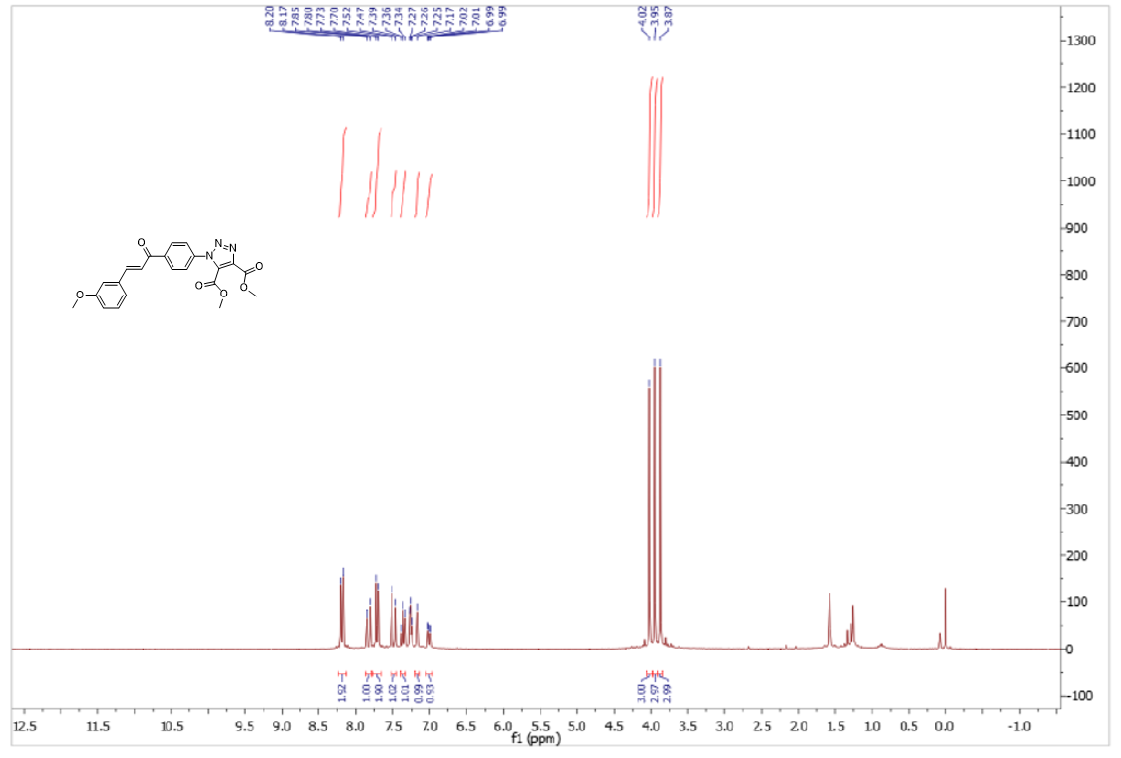


Figure S24. ^1^H NMR spectrum of Compound 6f


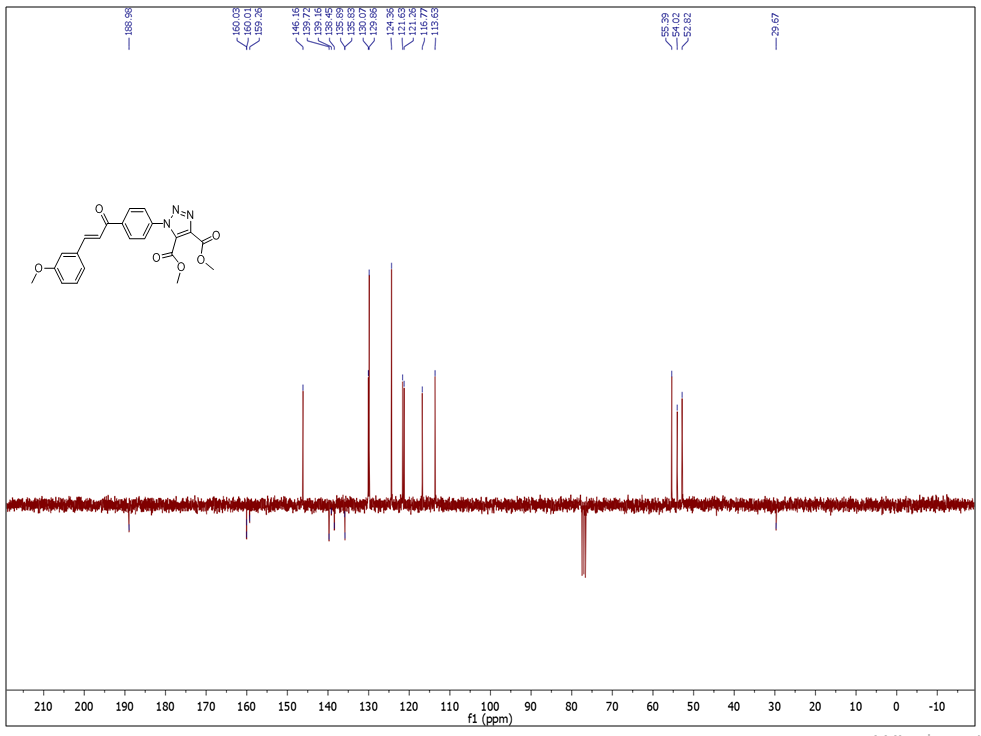


Figure S25. ^13^C-APT NMR spectrum of Compound 6f


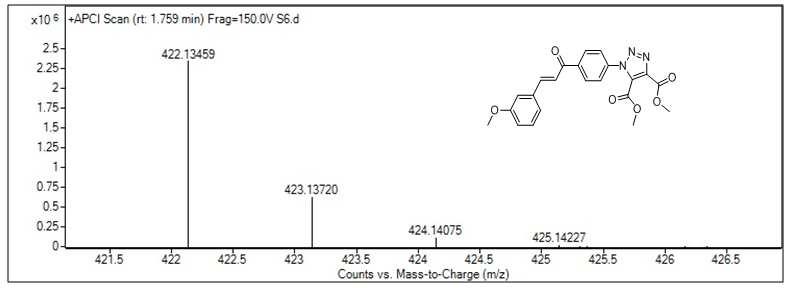


Figure S26. HRMS spectrum of Compound 6f


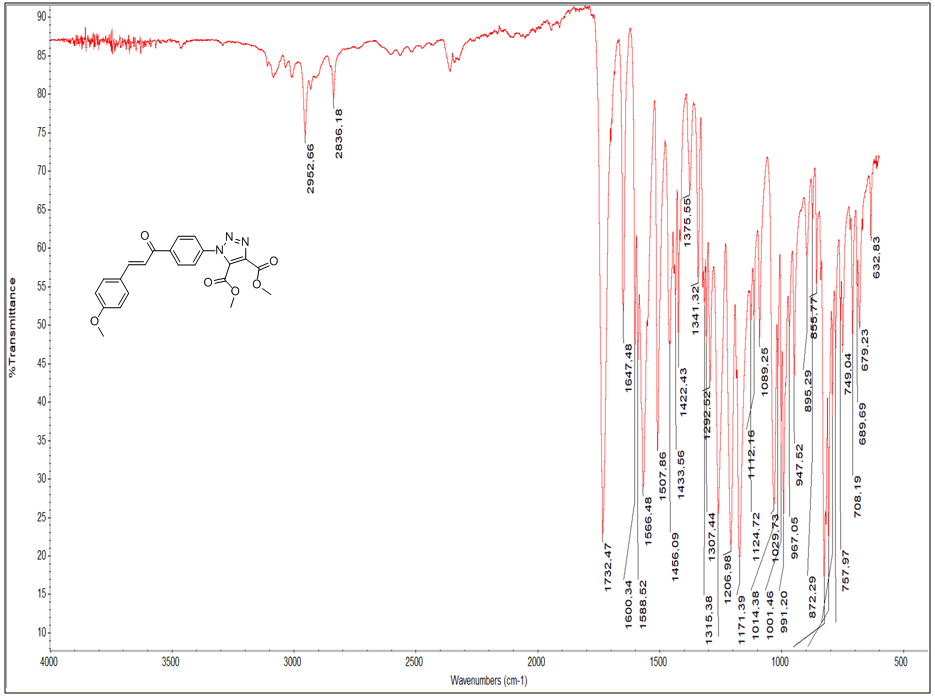


Figure S27. FTIR spectrum of Compound 6g


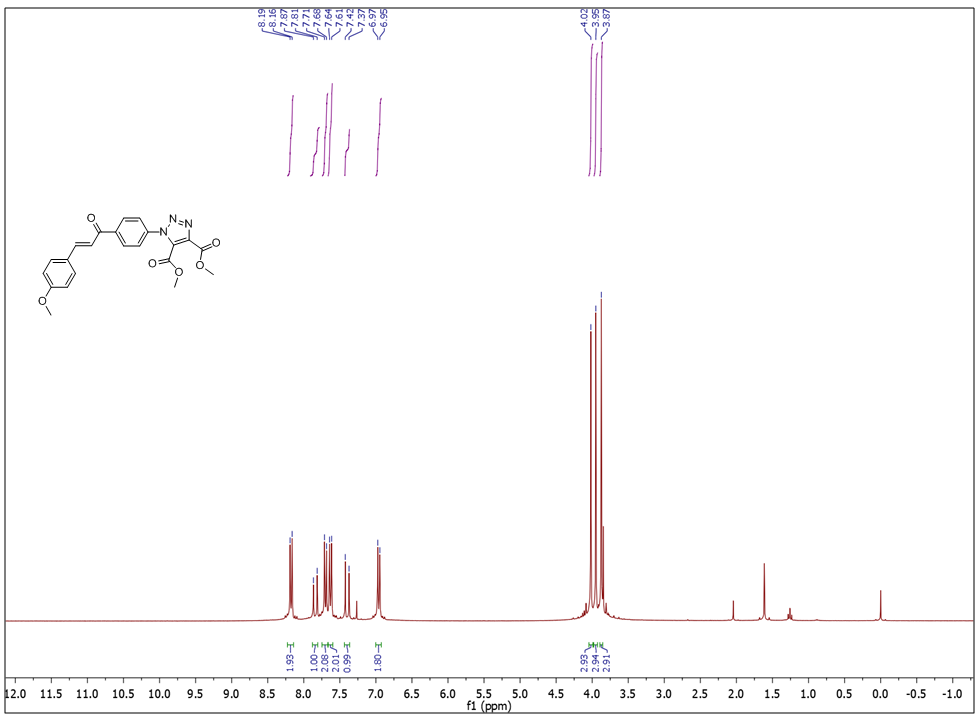


Figure S28. ^1^H NMR spectrum of Compound 6g


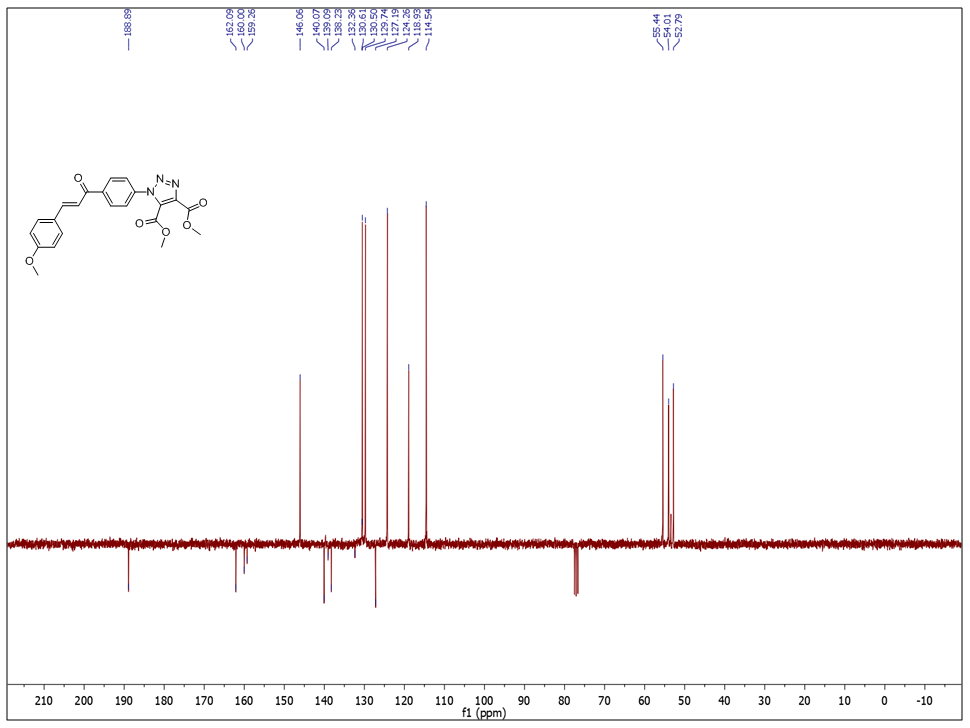


Figure S29. ^13^C-APT NMR spectrum of Compound 6g


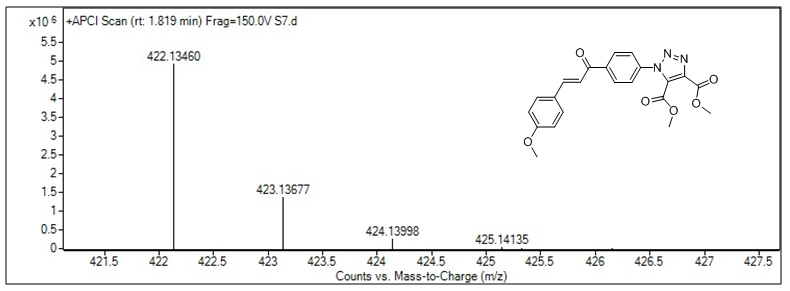


Figure S30. HRMS spectrum of Compound 6g


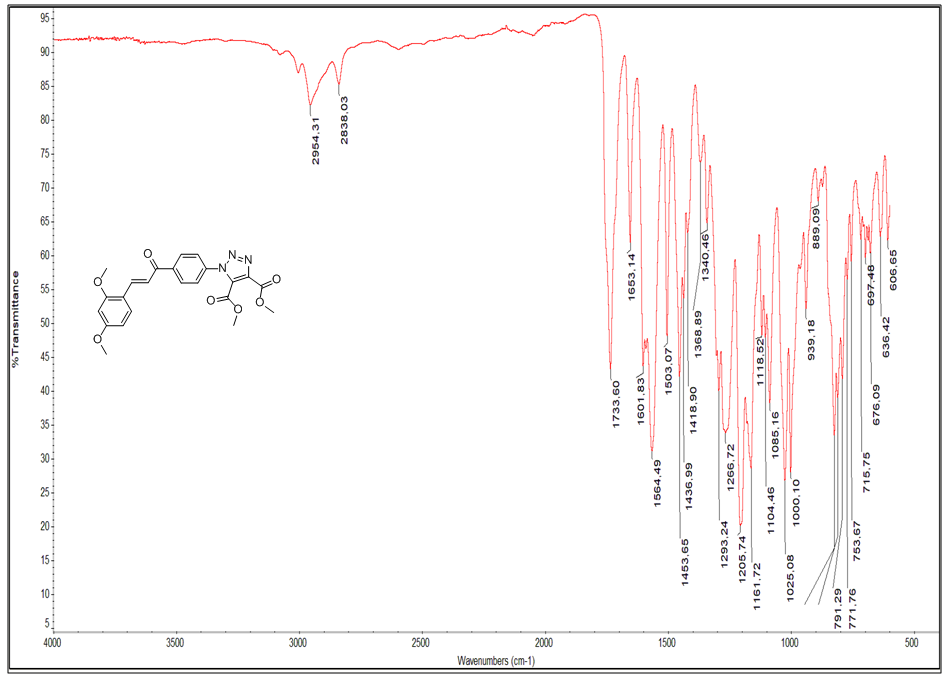


Figure S31. FTIRspectrum of Compound 6h


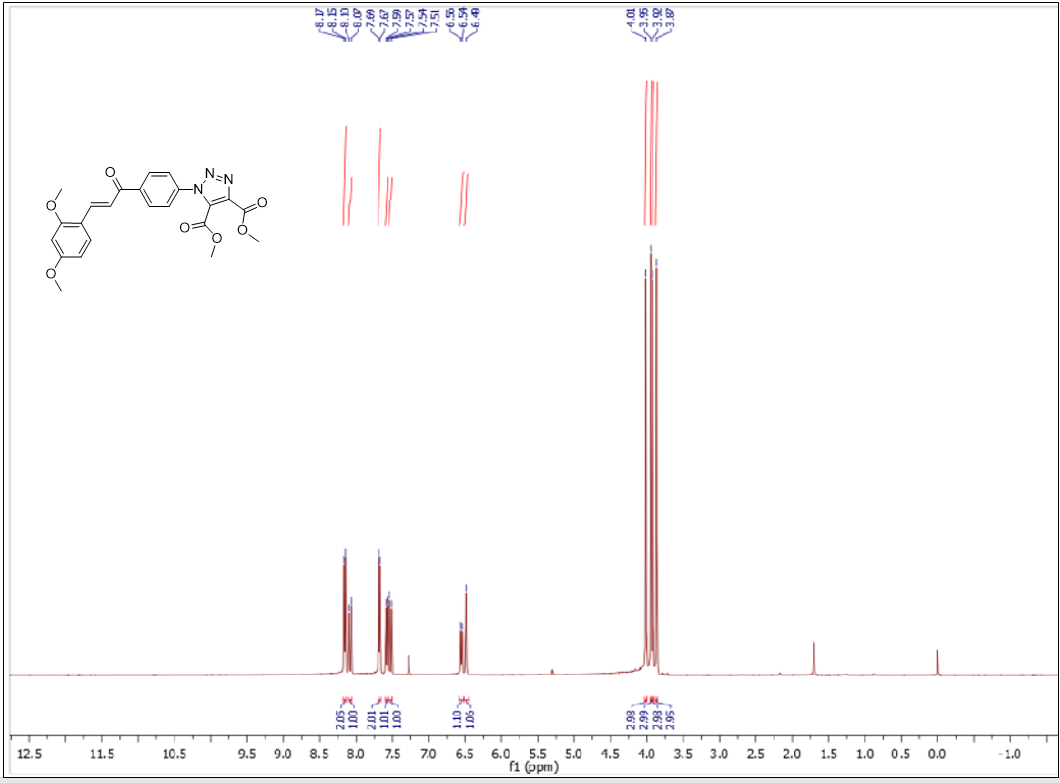


Figure S32. ^1^H NMR spectrum of Compound 6h


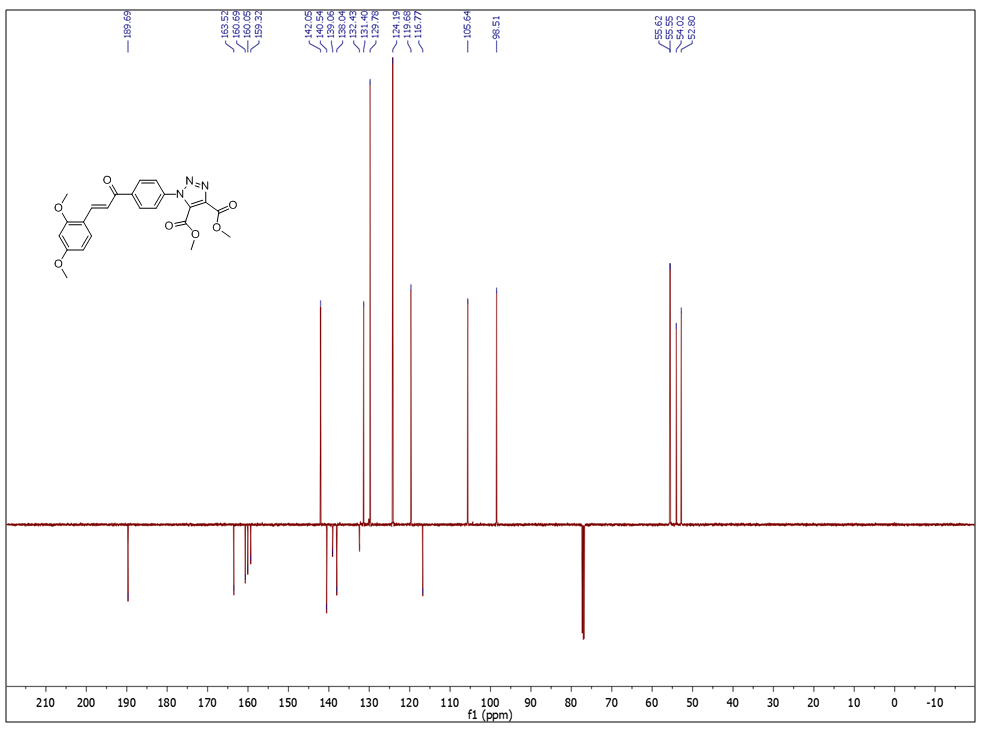


Figure S33. ^13^C-APT NMR spectrum of Compound 6h


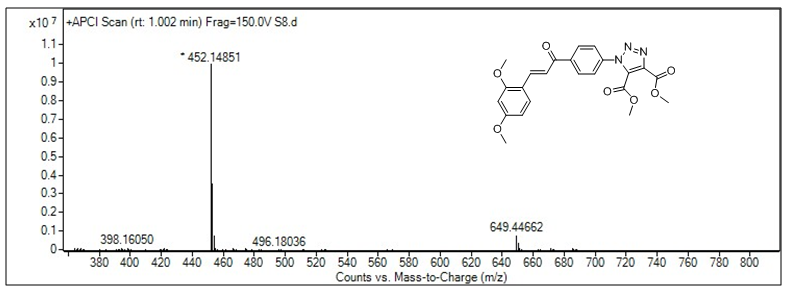


Figure S34. HRMS spectrum of Compound 6h


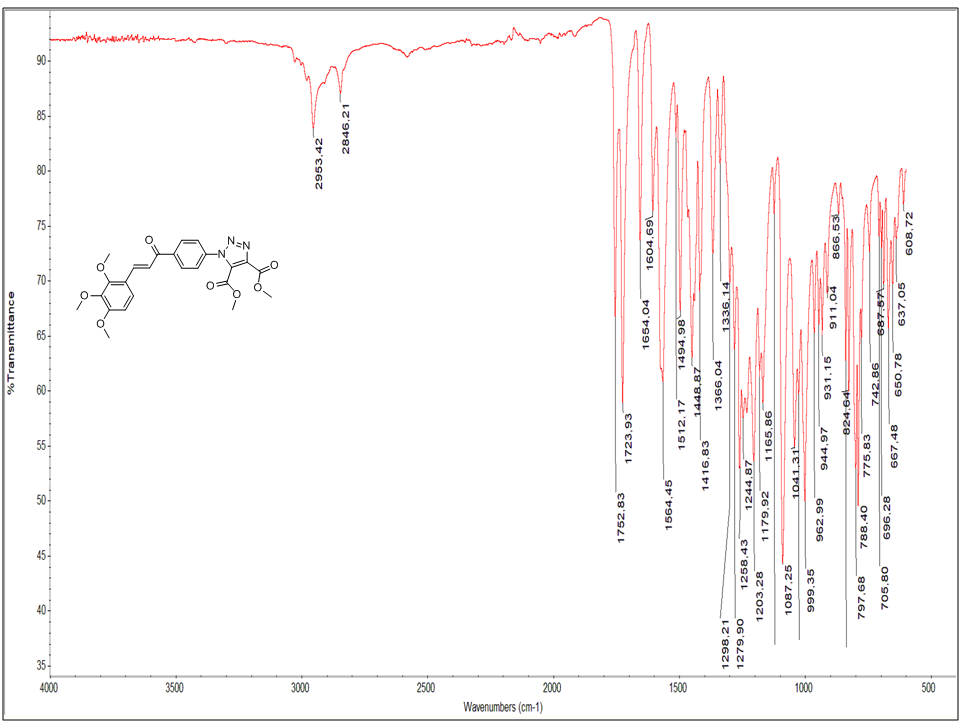


Figure S35. FTIR spectrum of Compound 6i


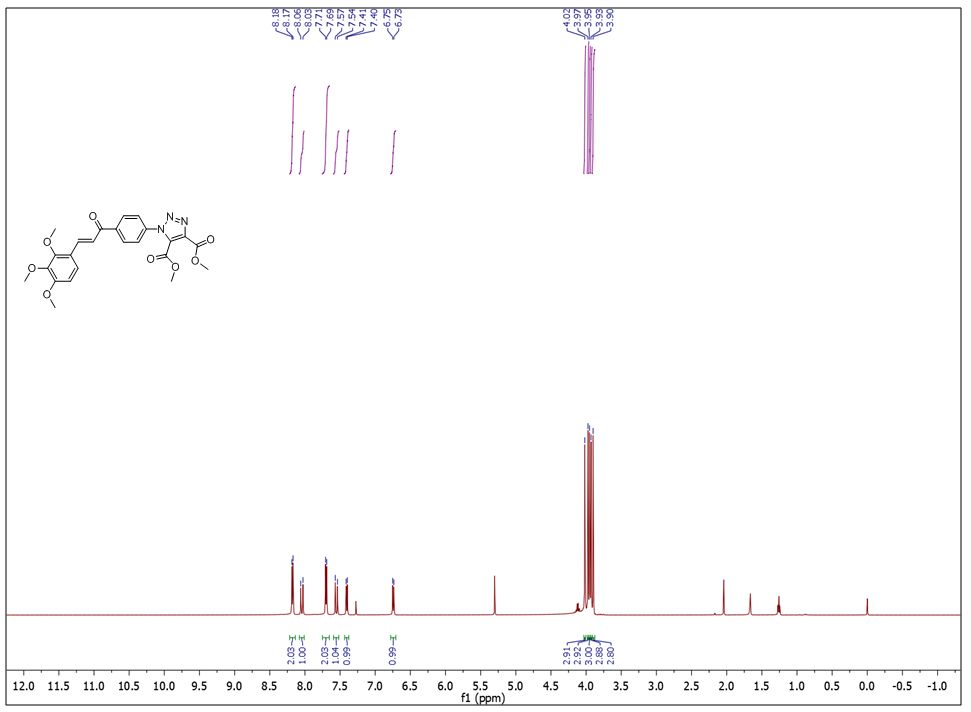


**Figure S36. ^1^H NMR** spectrum of Compound **6i**


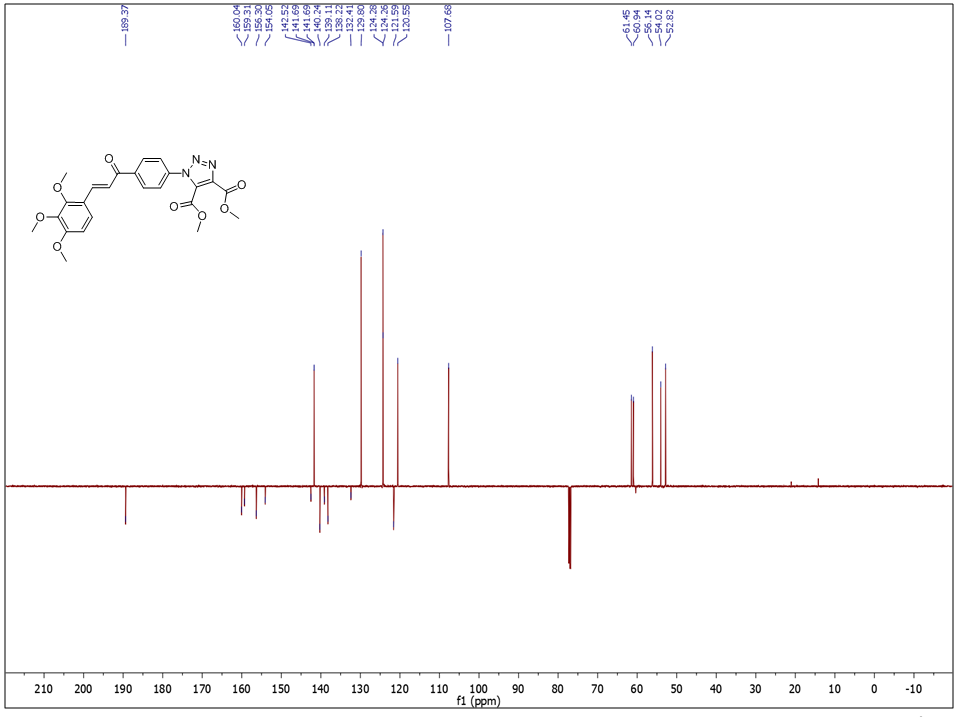


Figure S37. ^13^C-APT NMR spectrum of Compound 6i


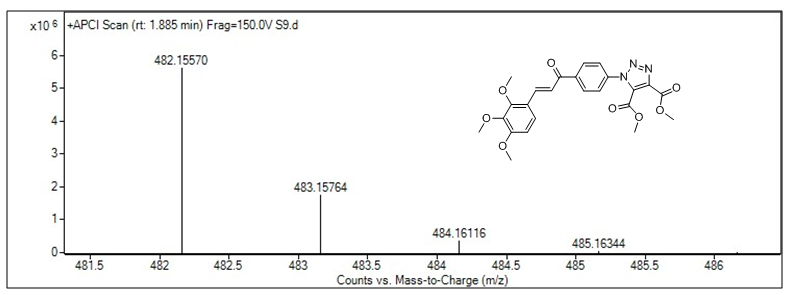


Figure S38. HRMS spectrum of Compound 6i

**4. References:**

[1] Buduma K, Kumar AN, Srinivas KVNS, Kumar JK, Chinde S, Domatti AK, et al. Synthesis and bioactivity evaluation of eugenol hybrids obtained by Mannich and 1,3 dipolar cycloaddition reactions. J Heterocyclic Chem. 2021;58(11):2078-89.

[2] Butler, C. R., Bendesky, J. and Schoffstall, A. M. (2021). Regioselective Reduction of 1H 1,2,3-Triazole Diesters. Molecules, 26(18), 26-42.

[3] Li, Y., et al., Discovery of orally active chalcones as histone lysine specific demethylase 1 inhibitors for the treatment of leukaemia. J Enzyme Inhib Med Chem, 2021. 36(1): p. 207-217.

[4] Zarghi, A., et al., Synthesis and biological evaluation of 1,3-diphenylprop-2-en-1-ones possessing a methanesulfonamido or an azido pharmacophore as cyclooxygenase-1/-2 inhibitors. Bioorg Med Chem, 2006. 14(20): p. 7044-50.

[5] Pingaew, R., et al., Synthesis, biological evaluation and molecular docking of novel chalcone coumarin hybrids as anticancer and antimalarial agents. Eur J Med Chem, 2014. 85: p. 65-76.

[6] Buduma K, Kumar AN, Srinivas KVNS, Kumar JK, Chinde S, Domatti AK, et al. Synthesis and bioactivity evaluation of eugenol hybrids obtained by Mannich and 1,3 dipolar cycloaddition reactions. J Heterocyclic Chem. 2021, 58(11), 2078-89.
