# Supplementary material for: 1,2,3‐Triazole‐Linked Chalcones as Privileged Scaffolds in Anticancer Agents
Source: Arch Pharm (Weinheim). 2026 Jul 7;359(6):e70291. doi: 10.1002/ardp.70291 (PMC13339972; doi:10.1002/ardp.70291)
Supplement: Supplementary file 2 — Supporting File 2 [file ARDP-359-e70291-s002.doc]

**Supplemental Material: Novel Compounds and Biological Screening Results**

**1,2,3-Triazole-Linked Chalcones as Privileged Scaffolds in Anticancer Agents**

Sümeyya Koldas^a,b^, Guler Yagiz Erdemir^a^, Ahsen Kilic^c,d^, Neslisah Barlak^c,d^, Omer Faruk Karatas^c,d^, and Aliye Altundas^a^*

^a^Gazi University, Faculty of Science, Department of Chemistry, 06560, Ankara, Turkiye

^b^Gazi University, Graduate School of Natural and Applied Sciences, 06560, Ankara, Turkiye

^c^Molecular Biology and Genetics Department, Erzurum Technical University, Erzurum, Turkiye

^d^Molecular Cancer Biology Laboratory, High Technology Application and Research Center, Erzurum Technical University, Erzurum, Turkiye

* Aliye Altundas

Gazi University, Faculty of Science, Department of Chemistry, 06560, Ankara, Turkiye

Email: [aaltundas@gazi.edu.tr](mailto:aaltundas@gazi.edu.tr)

| **Compound No.** | **InChI** | **Biological Activity (IC_50_)** |
| --- | --- | --- |
| 6a | InChI=1S/C21H17N3O5/c1-28-20(26)18-19(21(27)29-2)24(23-22-18)16-11-9-15(10-12-16)17(25)13-8-14-6-4-3-5-7-14/h3-13H,1-2H3/b13-8+ | IC_50_ (FaDu) >100 µM |
| 6b | InChI=1S/C21H16FN3O5/c1-29-20(27)18-19(21(28)30-2)25(24-23-18)15-10-7-14(8-11-15)17(26)12-9-13-5-3-4-6-16(13)22/h3-12H,1-2H3/b12-9+ | IC_50_ (FaDu) >100 µM |
| 6c | InChI=1S/C21H16FN3O5/c1-29-20(27)18-19(21(28)30-2)25(24-23-18)16-9-7-14(8-10-16)17(26)11-6-13-4-3-5-15(22)12-13/h3-12H,1-2H3/b11-6+ | IC_50_ (FaDu): 42.05 ± 6.23 µM  IC_50_ (A549): 30.382 ± 0.26 µM  IC_50_ (CaCo-2): 39.86 ± 3.9 µM  IC_50_ (PNT1a): 55.47±3.60 µM |
| 6d | InChI=1S/C21H16FN3O5/c1-29-20(27)18-19(21(28)30-2)25(24-23-18)16-10-6-14(7-11-16)17(26)12-5-13-3-8-15(22)9-4-13/h3-12H,1-2H3/b12-5+ | IC_50_ (FaDu) >100 µM |
| 6e | InChI=1S/C22H19N3O6/c1-29-18-7-5-4-6-15(18)10-13-17(26)14-8-11-16(12-9-14)25-20(22(28)31-3)19(23-24-25)21(27)30-2/h4-13H,1-3H3/b13-10+ | IC_50_ (FaDu): 79.88 ± 3.99 µM  IC_50_ (A549): 41.049 ± 8.18 µM  IC_50_(CaCo-2):47.095 ± 14.69µM  IC_50_ (PNT1a): 88.10±1.44 µM |
| 6f | InChI=1S/C22H19N3O6/c1-29-17-6-4-5-14(13-17)7-12-18(26)15-8-10-16(11-9-15)25-20(22(28)31-3)19(23-24-25)21(27)30-2/h4-13H,1-3H3/b12-7+ | IC_50_ (FaDu): 41.12 ± 9.14 µM  IC_50_ (A549): 11.46 ± 9.87 µM  IC_50_(CaCo-2): 19.88 ± 4.2 µM  IC_50_ (PNT1a): 88.66±7.63 µM |
| 6g | InChI=1S/C22H19N3O6/c1-29-17-11-4-14(5-12-17)6-13-18(26)15-7-9-16(10-8-15)25-20(22(28)31-3)19(23-24-25)21(27)30-2/h4-13H,1-3H3/b13-6+ | IC_50_ (FaDu) >100 µM |
| 6h | InChI=1S/C23H21N3O7/c1-30-17-11-7-15(19(13-17)31-2)8-12-18(27)14-5-9-16(10-6-14)26-21(23(29)33-4)20(24-25-26)22(28)32-3/h5-13H,1-4H3/b12-8+ | IC_50_ (FaDu) >100 µM |
| 6i | InChI=1S/C24H23N3O8/c1-31-18-13-9-15(21(32-2)22(18)33-3)8-12-17(28)14-6-10-16(11-7-14)27-20(24(30)35-5)19(25-26-27)23(29)34-4/h6-13H,1-5H3/b12-8+ | IC_50_ (FaDu) >100 µM |

^a^ Barlak N, Kusdemir G, Gumus R, Gundogdu B, Sahin MH, Tatar A, et al. Overexpression of POFUT1 promotes malignant phenotype and mediates perineural invasion in head and neck squamous cell carcinoma. Cell Biol Int. 2023.
